# Extensive gene rearrangements in the mitogenomes of congeneric annelid species and insights on the evolutionary history of the genus *Ophryotrocha*

**DOI:** 10.1186/s12864-020-07176-8

**Published:** 2020-11-23

**Authors:** Astrid Tempestini, Gloria Massamba-N’Siala, Fanny Vermandele, Nicholas Beaudreau, Mathieu Mortz, France Dufresne, Piero Calosi

**Affiliations:** Département de Biologie, Chimie et Géographie, Université du Québec à Rimouski, 300 Allée des Ursulines, Rimouski, QC, G5L 3A1 Canada

**Keywords:** Molecular phylogeny, Dorvilleidae, Mitogenome, Next generation sequencing, Model species, Reproductive mode

## Abstract

**Background:**

Annelids are one the most speciose and ecologically diverse groups of metazoans. Although a significant effort has been recently invested in sequencing genomes of a wide array of metazoans, many orders and families within the phylum Annelida are still represented by a single specimen of a single species. The genus of interstitial annelids *Ophryotrocha* (Dorvilleidae, Errantia, Annelida) is among these neglected groups, despite its extensive use as model organism in numerous studies on the evolution of life history, physiological and ecological traits. To compensate for the paucity of genomic information in this genus, we here obtained novel complete mitochondrial genomes of six *Ophryotrocha* species using next generation sequencing. In addition, we investigated the evolution of the reproductive mode in the *Ophryotrocha* genus using a phylogeny based on two mitochondrial markers (COXI and 16S rDNA) and one nuclear fragment (Histone H3).

**Results:**

Surprisingly, gene order was not conserved among the six *Ophryotrocha* species investigated, and varied greatly as compared to those found in other annelid species within the class Errantia. The mitogenome phylogeny for the six *Ophryotrocha* species displayed a separation of gonochoric and hermaphroditic species. However, this separation was not observed in the phylogeny based on the COX1, 16S rDNA, and H3 genes. Parsimony and Bayesian ancestral trait reconstruction indicated that gonochorism was the most parsimonious ancestral reproductive mode in *Ophryotrocha* spp.

**Conclusions:**

Our results highlight the remarkably high level of gene order variation among congeneric species, even in annelids. This encourages the need for additional mitogenome sequencing of annelid taxa in order to properly understand its mtDNA evolution, high biodiversity and phylogenetic relationships.

**Supplementary Information:**

**Supplementary information** accompanies this paper at 10.1186/s12864-020-07176-8.

## Background

Mitochondrial DNA (hereafter mtDNA) has been invaluable in the field of molecular evolution and phylogenetics, and is still a widely used marker today [1, 2]. Maternal inheritance and near absence of recombination have popularised its use in many eukaryotes [3]. Most of metazoan mitochondrial genomes are circular molecules that typically include 13 protein coding genes (PCG), two ribosomal RNA genes, 22 transfer RNA genes and a control region ([4], but see [5, 6]). The mitochondrial gene content is almost invariant among species, but the gene order has been found to vary considerably across Metazoans (such as flatworms, molluscs and tunicates [7]), generating interest in using mitochondrial DNA (mtDNA) gene order for phylogenetic inference [2]. The advent of next generation sequencing has made it easier to obtain mitochondrial genomes even for classical non-model organisms. This enables the detection of gene rearrangements, as well as phylogenetic relationships among and within diverse phyla [8]. Gene order is known to vary extensively within the phyla Mollusca [9], Arthropoda [10] and Annelida [11, 12].

Although over two thousand metazoan genomes have been sequenced to date [13], entire phyla such as Annelida have been widely neglected, with many orders or families often represented by a single specimen of a single species [14]. With more than 17,000 species described, annelids are among the most speciose and ecologically important groups of metazoans [15–17]. The extraordinary morphological and ecological diversity of annelids is only comparable to that of crustaceans and molluscs [18]. Furthermore, their exceptional plasticity and adaptability have enabled them to colonise all domains, from marine and freshwater to terrestrial habitats, evolving a wide variety of life history strategies, reproductive modes and feeding habits [19]. Marine annelids have been extensively used as model organisms for the investigation of central questions in ecotoxicology, ecology, physiology, development and evolution, owing to the fact that they are relatively easy to maintain and rear under laboratory conditions (reviewed in [20–22]). For example, the bristle worm *Capitella teleta* Blake, Grassle & Eckelbarger, 2009 (former *Capitella* sp. I) and the Dumeril’s clam worm *Platynereis dumerilii* (Audouin & Milne Edwards, 1833) have been primary biological systems for developmental, evolutionary and neurobiological studies [23–25], and are both part of a long list of annelids used as indicators for biomonitoring and eco-toxicology tests (reviewed in [26, 27]).

For similar reasons, the interstitial worms in the genus *Ophryotrocha* Claparède & Mecznikow, 1869 have been widely used to investigate the eco-evolution of functional traits (e.g. [28, 29]) and reproductive strategies (e.g. [30–32]), and more recently, to investigate species’ ability to tolerate, respond and adapt to global changes [33–38]. Although this group of annelids has been the focus of several descriptive and experimental studies over several decades (Additional file 1), our understanding of their genomics is scarce when compared to other annelids and marine invertebrate genera, thus limiting its potential as model system [22]. As new areas in the sea are explored, new *Ophryotrocha* species are regularly discovered and described [39–46], and with them the need to clarify the phylogenetic relationships within this genus. For example, recent molecular evidence is suggesting the presence of complexes or lineages in species that were originally considered as independent taxonomic units [42, 45]. Moreover, based on mitochondrial fragments of 16S and cytochrome oxidase I (COXI) genes, and nuclear H3 genes, a clear separation between gonochoric and hermaphroditic species has been proposed by multiple studies, with the hermaphroditism considered as the plesiomophic state for this genus [40, 41]. Providing additional genomic information on *Ophryotrocha* would certainly increase its usefulness as an emerging interdisciplinary model.

In order to improve our knowledge on the evolutionary history of the genus *Ophryotrocha*, we: (i) characterised for the first time the complete mitochondrial genomes of six *Ophryotrocha* species (*Ophryotrocha adherens* Paavo, Bailey-Brock & Akesson, 2000; *Ophryotrocha diadema* Åkesson, 1976; *Ophryotrocha japonica* Paxton & Åkesson, 2010; *Ophryotrocha labronica* La Greca & Bacci, 1962; *Ophryotrocha puerilis* Claparède & Mecznikow, 1869 and *Ophryotrocha robusta* Paxton & Åkesson, 2010), (ii) compared the gene orders of the six species with those described for the main annelids’ taxonomic groups, (iii) used these novel mitogenomes to investigate the phylogenetic relationships among the six corresponding *Ophryotrocha* species and, finally, (iv) updating the *Ophyrotrocha* phylogeny to portray how reproductive mode may be linked to evolutionary history in this genus.

## Results

### Sequencing

The number of pair-end reads obtained varied from 4,328,646 and 12,012,958 million (Table 1). We succeeded in circularising two mitochondrial genomes (*O. diadema* and *O. puerilis*). The other genomes were partial, but all 13 PCG were retrieved. The coverage for each mitochondrial genome or fragment varied between 65x (*O. labronica*) and 256x (*O. adherens*). The length of mtDNA genomes varied from 14,428 bp (*O. robusta*) to 15,941 bp (*O. puerilis*).

**Table 1:** Sequencing characteristics of the six *Ophryotrocha* species investigated

Species	Total Reads	Length (Bp)	Coverage	Comments
*O. adherens*	7,065,702	15,239	256	linear
*O. diadema*	8,242,992	15,796	207	circular
*O. japonica*	4,328,646	14,71	151	linear
*O. labronica*	5,850,626	15,981	65	linear
*O. puerilis*	7,181,522	15,941	181	circular
*O. robusta*	12,012,958	14,428	109	linear

### Genome organisation and features

#### Protein coding genes

**Fig. 1:**
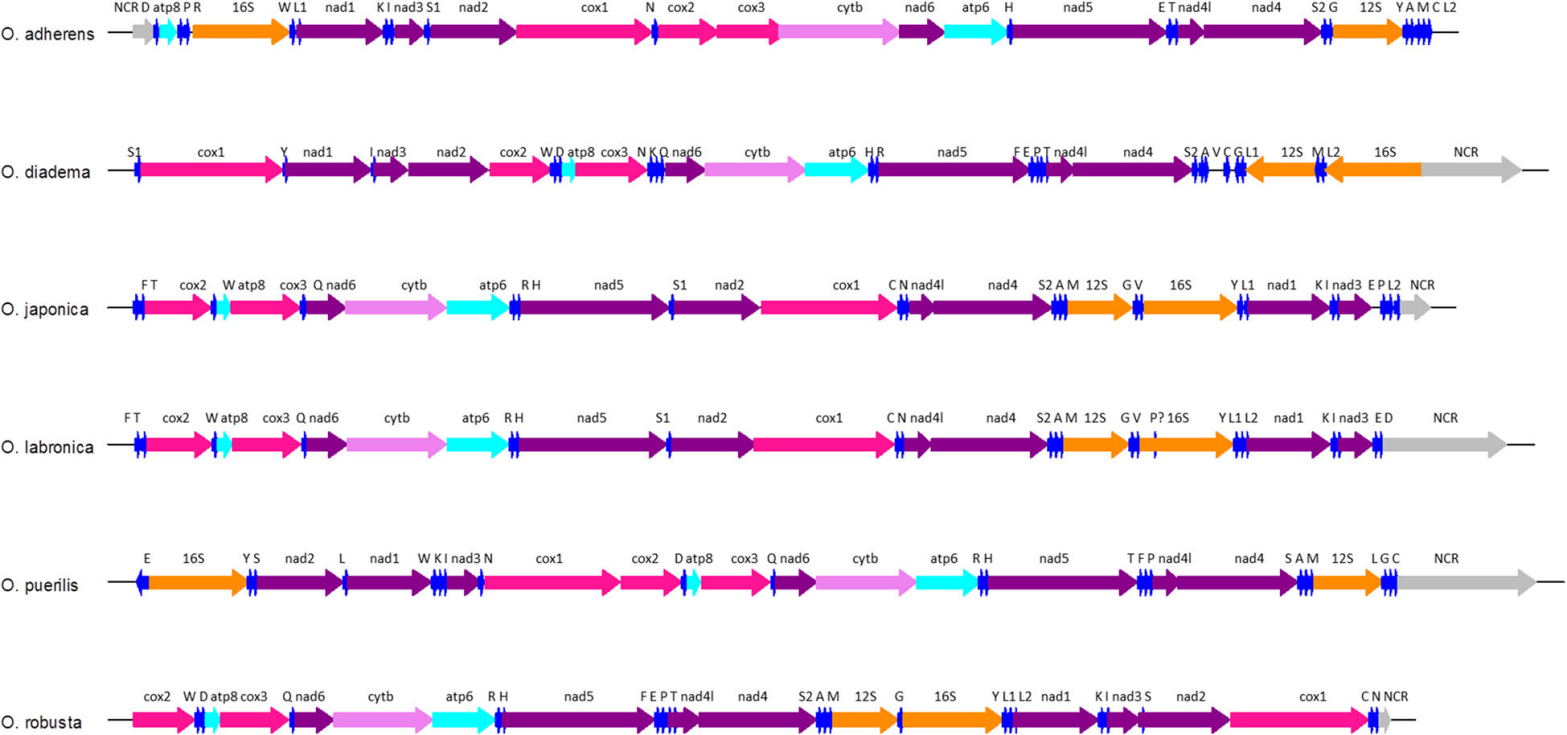
Mitochondrial genome of the six *Ophryotrocha* species. Colors represent gene complexes. Lightblue = ATP, Pink = cytochrome oxidase, lightpink = Cytochrome b, Purple = NADH, orange = ribosomal subunits, Blue = tRNA, Grey = Non coding region (NCR). The orientation of the arrows represents the orientation of the gene

We retrieved all the 13 PCG found in metazoans. All genes were coded on the plus strand, except for two ribosomal genes and some tRNAs of *O. diadema* that were found on the minus strand (Fig. 1, Additional files 2, 3, 4, 5, 6, and 7). Most of the genes started with the ATG codon and finished with the TAA stop codon (Table 2). Other alternative start codons were ATT, ATA, TTA and GTG. Ten codons were highly used in all species (Relative Synonymous Codon Usage (RSCU) > 1.25) and most of them were the type of NNU: UAU, GUU, ACU, AAU, UUU, AUA, CUC, AUU, GAU, GCU. Eleven codons were avoided in all species (RSCU < 0.75): UCG, UAC, GUC, ACG, CCG, UUA, CUA, AUC, GGC, UCC and GAC (Table 2). Valine (GUA) was mostly used in *O. adherens*, *O. diadema* and *O. puerilis*. Serine codons (AGU and AGG) were highly biased in *O. japonica*, *O. labronica* and *O. robusta*, while Threonine codon (ACA) was biased in *O. labronica* and *O. puerilis*. Regarding cysteine and glutamic acid, codon usage frequency (UGU and GAG) was higher in *O. japonica*, *O. labronica* and *O. robusta*. The AT content was high in the genome of all six *Ophryotrocha* species and ranged between 64.1% (*O. japonica*) and 71.5% (*O. adherens*). In particular, *O. adherens*, *O. diadema* and *O. robusta* harboured the highest AT content. Most of the PCG in each *Ophryotrocha* species harboured negative AT-skew (Table 3), suggesting a bias in T, with the exception of seven PCG in *O. puerilis*, which held a positive value of AT-skew. Positive values of AT-skew were observed in the ribosomal genes of five *Ophryotrocha* species, indicating a bias in A. In addition, negative AT-skew was observed for the ribosomal genes of *O. diadema*, indicating a bias in T. All PCG in *O. adherens*, *O. diadema* and *O. puerilis* had negative values of GC-skew suggesting a bias in C, whereas positive GC-skew was found for all PCG of *O. japonica*, *O. labronica* and *O. robusta*.

**Table 2:** Codon usage in the 13 PCG for each of the six *Ophryotrocha* species investigated

RSCU							
**AA**	**Codon**	***O. adherens***	***O. diadema***	***O. japonica***	***O. labronica***	***O. puerilis***	***O. robusta***
STOP	UAG	0.514	0.536	0.902	0.916	0.653	0.73
	UAA	**1.486**	**1.464**	1.098	1.084	**1.347**	**1.27**
Ala	GCU	**1.905**	**1.414**	**2.099**	**1.942**	**1.401**	**1.923**
	GCG	0.229	0.241	0.673	0.816	0.613	0.538
	GCC	0.762	1.172	0.436	0.466	0.964	0.769
	GCA	1.105	1.172	0.792	0.777	1.022	0.769
Cys	UGU	1.215	1.211	**1.667**	**1.469**	1.091	**1.488**
	UGC	0.785	0.789	0.333	0.531	0.909	0.512
Asp	GAU	**1.354**	**1.419**	**1.588**	**1.481**	**1.288**	**1.6**
	GAC	0.646	0.581	0.412	0.519	0.712	0.4
Glu	GAG	0.383	0.563	**1.3**	**1.244**	0.69	**1.299**
	GAA	**1.617**	**1.438**	0.7	0.756	**1.31**	0.701
Phe	UUU	**1.398**	**1.296**	**1.721**	**1.734**	**1.333**	**1.739**
	UUC	0.602	0.704	0.279	0.266	0.667	0.261
Gly	GGU	0.991	0.76	**1.384**	**1.253**	1.216	1.058
	GGG	1.101	1.16	**1.676**	**1.937**	**1.324**	**1.935**
	GGC	0.404	0.44	0.368	0.241	0.649	0.413
	GGA	**1.505**	**1.64**	0.573	0.57	0.811	0.594
His	CAC	0.703	0.869	0.75	0.44	1.119	0.563
	CAU	**1.297**	1.131	**1.25**	**1.56**	0.881	**1.438**
Ile	AUU	**1.561**	**1.343**	**1.689**	**1.726**	**1.357**	**1.691**
	AUC	0.439	0.657	0.311	0.274	0.643	0.309
Arg	AAA	**1.514**	**1.72**	1	1.092	**1.373**	**1.315**
	AAG	0.486	0.28	1	0.908	0.627	0.685
Leu	UUG	1.109	**1.574**	1.104	**1.252**	**1.435**	1.097
	UUA	0.66	0.503	0.466	0.417	0.609	0.602
	CUA	0.547	0.529	0.687	0.712	0.609	0.581
	CUC	**1.684**	**1.394**	**1.742**	**1.62**	**1.348**	**1.72**
	CUG	**1.58**	**1.555**	1.17	**1.268**	**1.381**	**1.346**
	CUU	0.42	0.445	0.83	0.732	0.619	0.654
Met	AUG	0.589	0.477	0.763	0.706	0.579	0.683
	AUA	**1.411**	**1.523**	**1.238**	**1.294**	**1.421**	**1.317**
Asn	AAC	0.531	0.612	0.368	0.321	0.764	0.452
	AAU	**1.469**	**1.388**	**1.632**	**1.679**	**1.236**	**1.548**
Pro	CCU	**1.333**	**1.347**	**1.347**	**2.108**	1.077	**1.509**
	CCG	0.4	0.166	0.408	0.387	0.418	0.264
	CCC	**1.28**	**1.326**	**1.429**	0.602	**1.341**	**1.547**
	CCA	0.987	1.161	0.816	0.903	1.165	0.679
Gln	CAG	0.667	0.667	0.727	0.824	0.66	1.051
	CAA	**1.333**	**1.333**	**1.273**	1.176	**1.34**	0.949
Arg	CGA	**1.957**	1.079	0.48	1	1.023	**1.277**
	CGC	0.34	**1.46**	0.32	0.462	1.023	0.34
	CGG	1.106	0.698	**2.32**	1.077	0.977	**1.362**
	CGU	0.596	0.762	0.88	**1.462**	0.977	1.021
Ser	AGC	0.725	0.843	0.503	0.536	1.051	0.655
	AGA	1.181	1.038	0.984	0.893	1.137	**1.311**
	UCA	**1.285**	**1.47**	0.721	0.734	**1.351**	0.829
	UCC	0.891	**1.232**	0.699	0.675	0.622	0.655
	UCG	0.269	0.411	0.306	0.139	0.279	0.27
	UCU	**2.093**	**1.686**	**1.333**	**1.27**	1.158	**1.311**
	AGG	0.767	0.692	**1.989**	**2.045**	1.072	**1.735**
	AGU	0.788	0.627	**1.464**	**1.707**	**1.33**	**1.234**
Thr	ACA	0.976	1.107	0.926	**1.31**	**1.373**	1.008
	ACC	0.78	0.929	0.778	0.724	0.974	0.773
	ACG	0.371	0.321	0.481	0.379	0.399	0.471
	ACU	**1.873**	**1.643**	**1.815**	**1.586**	**1.255**	**1.748**
Val	GUC	0.374	0.44	0.251	0.362	0.429	0.488
	GUG	0.604	0.734	1.103	0.993	0.667	0.882
	GUU	**1.295**	**1.394**	**1.743**	**1.678**	**1.548**	**1.795**
	GUA	**1.727**	**1.431**	0.903	0.966	**1.357**	0.835
Trp	UGA	**1.4**	**1.302**	0.93	0.896	1.209	0.743
	UGG	0.6	0.698	01.07	1.104	0.791	**1.257**
Tyr	UAC	0.599	0.717	0.453	0.453	0.562	0.479
	UAU	**1.401**	**1.283**	**1.547**	**1.547**	**1.438**	**1.521**

Numbers represent the relative synonymous codon usage (RSCU). *AA* Amino acids. The amino acids more frequently used are in bold (RSCU > 1.25). Underlined numbers indicate the avoided amino acids

**Table 3:** AT content and AT/GC skew of the mitochondrial genomes of the six *Ophryotrocha* species investigated

Name	***O. adherens***			***O. diadema***			***O. japonica***			***O. labronica***			***O. puerilis***			***O. robusta***		
	AT content %	AT skew	GC skew	AT content %	AT skew	GC skew	AT content %	AT skew	GC skew	AT content %	AT skew	GC skew	AT content %	AT skew	GC skew	AT content %	AT skew	GC skew
atp6	71.6	−0.179	−0.364	66.4	−0.096	−0.381	64	−0.291	0.296	66.9	−0.262	0.390	63.6	0.006	−0.222	66.3	−0.225	0.231
atp8	69.6	−0.124	−0.322	69.1	0.036	−0.333	66.7	−0.184	0.407	68.5	−0.101	0.401	66	0.185	−0.094	66.7	−0.019	0.285
cox1	66.7	−0.109	−0.036	62	−0.035	−0.137	60.8	−0.273	0.199	64.4	−0.242	0.258	58	−0.103	−0.048	63	−0.203	0.205
cox2	67.5	−0.096	−0.095	65.8	−0.036	−0.222	60.3	−0.207	0.390	63.4	− 0.174	0.404	62.6	0.08	− 0.091	64.4	− 0.127	0.208
cox3	64.8	−0.173	− 0.111	61.2	− 0.144	− 0.080	59.3	− 0.305	0.417	60.4	− 0.278	0.394	59.4	− 0.087	− 0.057	62.3	− 0.236	0.252
cob	67.4	− 0.125	− 0.098	64.7	− 0.054	− 0.235	60.2	− 0.272	0.330	64	− 0.288	0.272	62.9	−0.091	− 0.097	65	− 0.185	0.135
nad1	71.4	−0.188	−0.140	68.6	−0.050	− 0.344	62.8	− 0.197	0.312	64.8	−0.201	0.239	65.4	0.015	−0.208	65.4	−0.196	0.237
nad2	76.4	−0.202	−0.322	74	−0.046	− 0.362	63.8	− 0.263	0.508	69.6	−0.201	0.461	66.1	0.035	−0.198	66.9	−0.154	0.352
nad3	75.7	−0.266	−0.207	65.6	−0.229	− 0.273	62.1	− 0.250	0.468	63.2	−0.199	0.321	66.4	−0.105	−0.196	68.3	−0.253	0.544
nad4	71.3	−0.130	−0.203	68.9	−0.068	− 0.395	64.1	− 0.242	0.376	65.7	−0.248	0.430	64.5	0.042	−0.144	66.5	−0.221	0.307
nad4L	73.5	−0.113	−0.192	68.5	−0.063	− 0.086	69.8	− 0.266	0.649	74.2	−0.259	0.643	66.7	−0.130	−0.081	69.2	−0.182	0.316
nad5	72.2	−0.105	−0.234	70.2	−0.034	− 0.356	63.9	− 0.208	0.396	66.4	−0.217	0.369	65.9	−0.011	−0.126	66.2	−0.160	0.349
nad6	76	−0.174	−0.267	75	−0.083	− 0.352	67.1	− 0.306	0.538	67.1	−0.285	0.488	66.8	0.003	−0.160	68.3	−0.262	0.401
srRNA	73.2	0.087	−0.082	69	−0.012	0.206	66.5	0.029	0.251	67.4	0.039	0.221	68.4	0.207	0.013	68.2	0.079	0.170
lrRNA	75	0.021	−0.016	70.2	−0.037	0.251	69.5	0.027	0.272	69.5	0.022	0.259	69.4	0.138	0.003	72.4	0.052	0.268
total	71.5	−0.099	−0.151	69.1	−0.025	− 0.243	64.3	− 0.185	0.358	66.3	−0.155	0.337	64.1	0.029	−0.092	66.8	−0.141	0.265

The highest variability in gene length was observed for NAD4, whose size ranged from 1239 bp (*O. labronica*) to 1341 bp (*O. diadema* and *O. adherens*). Cytochrome b in *O. adherens* was the longest compared to the other *Ophryotrocha* species. Several overlaps and small non-coding regions between tRNA and genes, or between two adjacent genes, were observed in all species and showed sizes that ranged from a few to hundreds of bases (Additional files 2, 3, 4, 5, 6, and 7).

#### rRNA and tRNA

The length of the small ribosomal unit varied between 736 bp (*O. japonica*) and 794 bp (*O. adherens*), while that of the large ribosomal subunit ranged between 1064 bp (*O. labronica*) and 1140 bp (*O. puerilis*). The lowest GC content was observed in *O adherens* for both small and large ribosomal units (24 and 20%, respectively). *Ophryotrocha japonica* harboured the highest GC content for both ribosomal units: 32% for 12S and 30% for 16S.

In *O. labronica* and *O. diadema*, we retrieved 22 tRNA genes based on their secondary leaf structure and their anticodon (see Additional files 8, 9, 10, 11, 12 and 13). In the remaining *Ophryotrocha* species, Valine and Glutamate were not found, while Aspartate was missing only in *O. japonica*. In *O. labronica*, tRNA-Pro was not completely recovered.

### Gene rearrangement

**Fig. 2:**
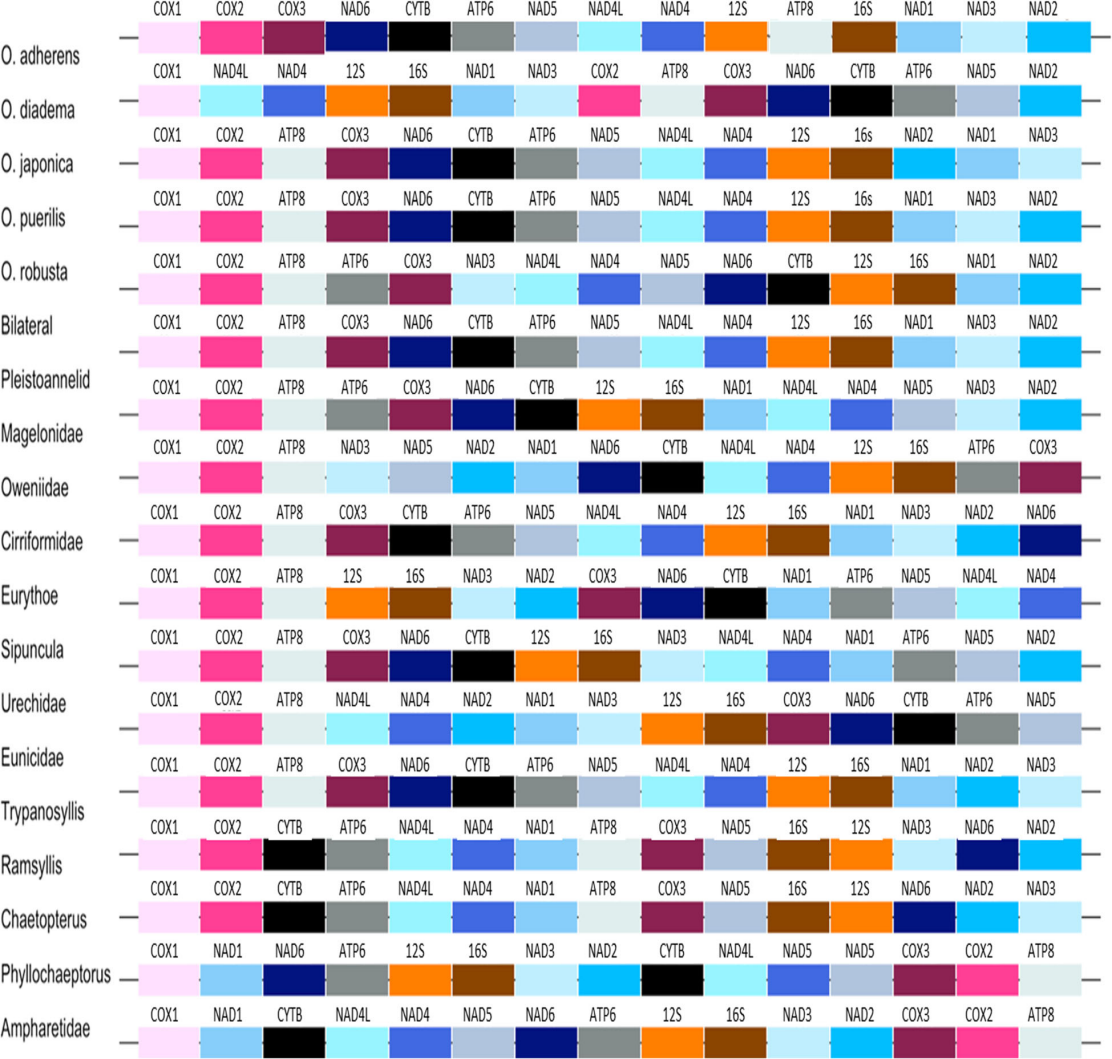
PCG gene order and ribosomal RNAs of the *Ophryotrocha* species studied and other annelids. Since *O. labronica* has the same PCG order as *O. japonica*, only the gene order of the latter was displayed. Each gene is represented by a specific color. The up and down blocks represent the position on the plus or the minus strand of the gene

The gene order varied widely among *Ophryotrocha* species (Fig. 2). The higher level of similarity between gene order was found between *O. robusta* and *O puerilis*, while the highest dissimilarity was observed between *O. japonica* and the two species *O. adherens* and *O. diadema* (Additional file 14). *Ophryotrocha japonica* and *O. labronica*, showed the same gene order, and differed from *O. robusta* by a single transposition of several genes from COX2 to NAD5. *Ophryotrocha robusta* and *O. puerilis* differed by a transposition of NAD2. *Ophryotrocha adherens* and *O. robusta* differed by a transposition of ATP8. *Ophryotrocha robusta* and *O. diadema* differed by two transpositions of NAD5 and the block of NAD1, NAD2, NAD3 and a reversion of the two ribosomal genes. *Ophryotrocha puerilis* and *O. adherens* differed by two transpositions of ATP8 and NAD2. *Ophryotrocha adherens* and *O. diadema* differed by three transpositions of NAD5, ATP8 and the block of NAD1, NAD2 and NAD3 genes, and a reversion of the ribosomal genes. *Ophryotrocha adherens* and *O. japonica* differed by a tandem duplication random loss of ATP8 and a transposition of the 16S, NAD1, NAD3. The gene order of *O. puerilis* was the most similar to that found in Eunicidae and Pleistoannelid (number of common interval = 91, Additional file 15), differing only by the position of NAD2 (Fig. 2). *Ophryotrocha diadema* and the Ampharetidae gene order were similar and differed by the position of NAD1, NAD2, NAD3 and the reversal of the two ribosomal genes. The gene order pattern of Pleistoannelida was similar to the one found in *O. adherens* and differed only by the transposition of ATP8. *Ophryotrocha robusta* had a gene order very similar to the Pleistoannelida differing only by a single transposition between NAD4 and NAD4L. The Errantia pattern with *O. japonica* and *O. labronica* gene order differed by the transposition of the COX2, ATP8, COX3, NAD6, CYTB, ATP6 and NAD5 regions. *Ophryotrocha robusta* gene order did not differ from that of Errantia. Magelonidae was closest to the putative bilateral pattern differing by several reversals of NAD3, NAD4L, NAD4, NAD5, NAD6, CYTB, ribosomal genes and NAD1. It also differed from the Lophotrochozoa pattern by several transpositions of COX3, NAD6 and CYTB.

### Phylogeny

#### *Mitochondrial phylogeny of the six studied* Ophryotrocha *species*

**Fig. 3:**
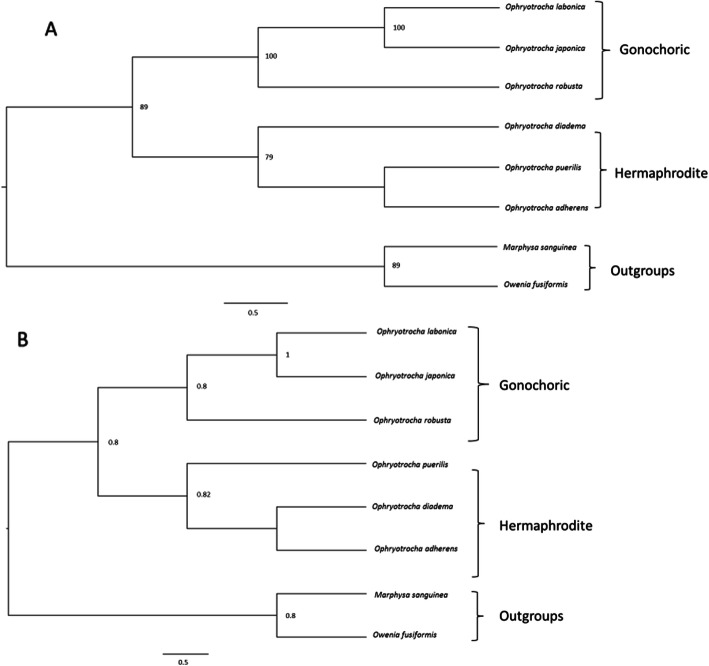
Phylogenetic trees based on concatenated mitochondrial PCG nucleotides under the K3P model with invariant gamma sites. **a**: ML tree. The number represents the aLTR obtained by maximum likelihood. Only LTR values greater than 70 are shown. **b**: BI tree. The number represents the posterior probability (pp). Only posterior probabilities greater than 0.7 are shown

The concatenation of the amino-acid sequences resulted in a fragment of 4098 residues. After removing the poorly aligned positions, 2242 residues were kept for phylogenetic analyses. The concatenation of the nucleotide sequences resulted in a fragment of 12,297 bp. After removing the poorly aligned positions, 6726 bp were kept for phylogenetic analyses. Bayesian and maximum likelihood amino-acid and nucleotide phylogenies were mostly congruent (Fig. 3). The six *Ophryotrocha* species clustered into two groups: one including the gonochoric species *O. labronica*, *O. japonica* and *O. robusta* and a second group including the hermaphroditic species *O. adherens*, *O. puerilis* and *O. diadema*. Only the position of *O. puerilis* within the hermaphrodite clade changes between ML and BI phylogenies.

#### Ophryotrocha *genus phylogeny and ancestral state reconstruction of the reproductive mode*

**Fig. 4:**
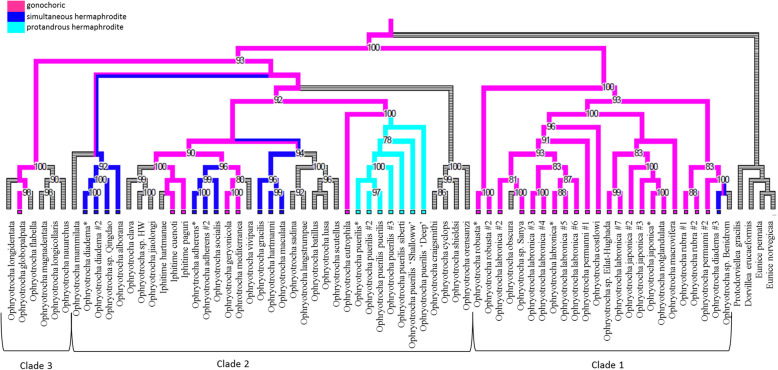
Maximum likelihood phylogeny of the genus *Ophryotrocha* based on the fragment of COXI, 16S and H3 under the GTR model with invariant gamma sites and ancestral state reconstruction. Values represent the aLTR support and the posterior probabilities, respectively. Only values greater than 70 are shown. * represents the species we used in this study. Branch color represents the reproductive mode for all the species and their common ancestors: light blue (protandrous hermaphrodite), dark blue (simultaneous hermaphrodite), pink (gonochoric) and grey for unknown mode

**Fig. 5:**
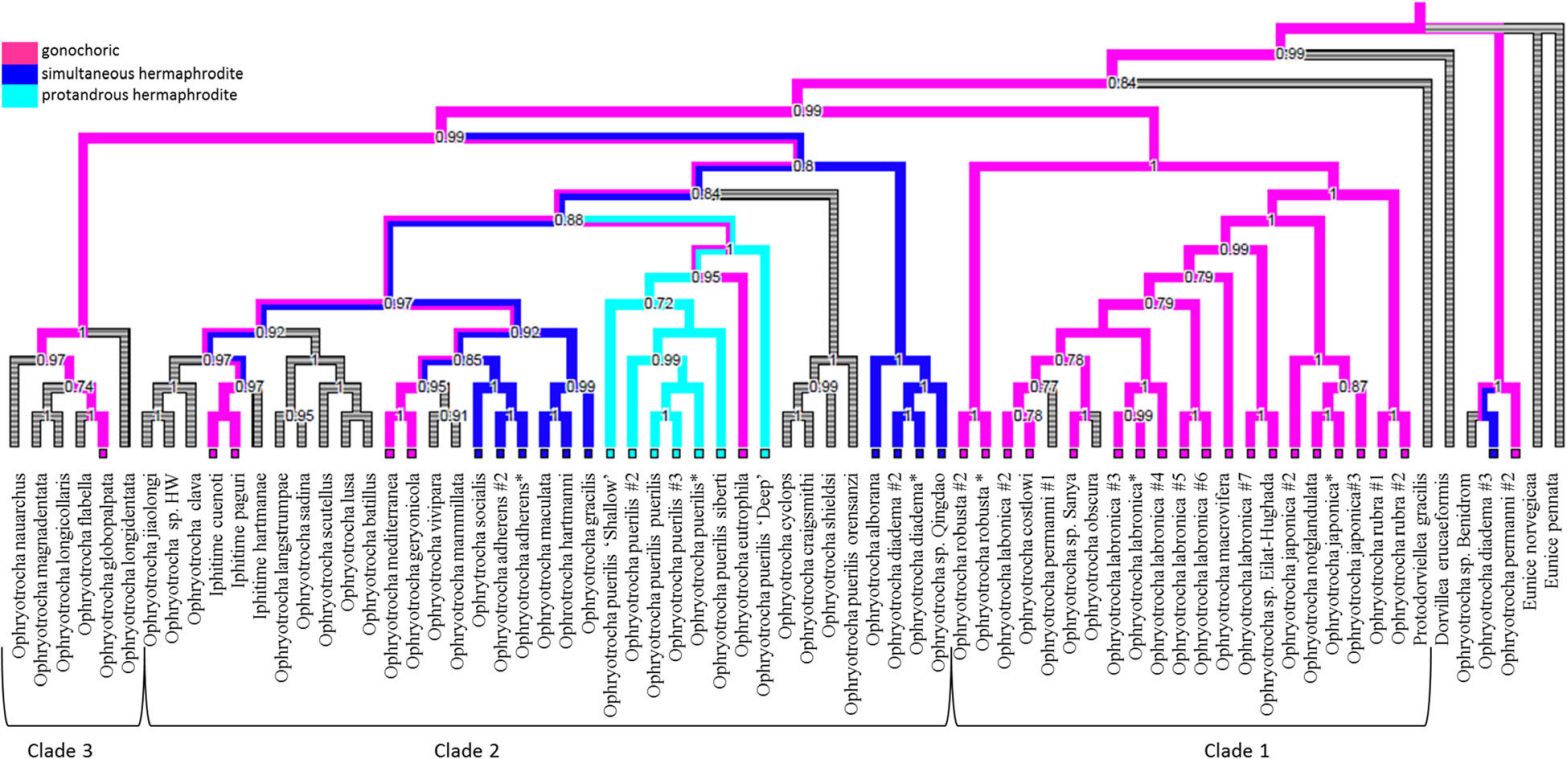
Bayesian phylogeny of the genus *Ophryotrocha* based on the fragments of COXI, 16S and H3 under the GTR model with invariant gamma sites and ancestral state reconstruction. Values represent the aLTR support and the posterior probabilities, respectively. Only posterior probabilities greater than 0.7 are shown. * represents the species we used in this study. Colors of the branch represent the reproductive mode for all the species and their common ancestors: light blue (protandrous hermaphrodite), dark blue (simultaneous hermaphrodite), pink (gonochoric) and grey for unknown mode

Both Bayesian inference (BI) and Maximum likelihood (ML) trees were mostly congruent and two major clades were found, i.e. clade 1 and 2, which displayed high support (aLTR = 100, pp. = 0.99) (Fig. 4 and Fig. 5). In both phylogenies, clade 1 was composed mostly of gonochoric species, except for the hermaphroditic species *O. diadema* #3 (EF464534). Clade 2 contained species with all three types of reproductive mode: such as *O. adherens* and species of the *O. puerilis* group, which are respectively simultaneous and protandrous hermaphroditic species, and some gonochoric species such as *Iphitime cuenoti*, *Iphitime paguri*, *Ophryotrocha geryonicola* and *Ophryotrocha globopalpata*. A third minor clade, clade 3, containing *O. nauarchus*, *O. globopalpata, O. flabella*, and *O. longidentata*, was identified in both phylogenies. Within clade 3, only the reproductive mode of *Ophryotrocha globopalpa*ta is known to be gonochorism. In the BI phylogeny, the species *O. diadema* EF464534, *O. permanni* EF464535 and *Ophryotrocha* sp. Benidorm were grouped together, but not included in a specific clade as observed in the ML phylogeny (aLTR = 83).

In the phylogeny of the *Ophryotrocha* genus, we identified 25 gonochoric species, seven protandrous hermaphrodite species and 11 simultaneous hermaphrodite species. All ancestral reproductive mode analyses suggest gonochorism as the reproductive mode for the last common ancestor of *Ophryotrocha* spp. (Figs. 4, 5, additional file 17). However, for the protandrous group containing all the *Ophryotrocha puerilis* species and the gonochoric species *Ophryotrocha eutrophila*, the support of the ancestral state was low (pp < 0.7), suggesting that the reproductive mode for the ancestor is not fully supported.

## Discussion

Our work is the first to report gene rearrangements involving protein coding genes (PCG) among annelid species belonging to the same genus. Also, in contrast to other known annelid mitochondrial genomes, we show that *Ophryotrocha diadema* possesses the ribosomal genes encoded on the minus strand. Finally, the ancestral state reconstruction of the reproductive mode shows that gonochorism is the plesiomorphic condition in the *Ophryotrocha* genus, not hermaphroditism as previously hypothesized. Below we discuss in detail these major findings, and their implications for understanding the molecular evolution of the mitochondrial genome within the genus *Ophryotrocha.*

### *Genome organisation and features of the six* Ophryotrocha *species*

We provide here the complete mitochondrial genome sequences for six *Ophryotrocha* species. The features of these mitogenomes did not differ from those reported from other annelid species. Their AT-content, AT and GC-skew, the length of the ribosomal genes and the initiative codons of the PCG are congruent with those reported for other annelids [18, 47–54] and metazoans [55]. GC content and GC-skew have an influence on the usage of codon and proteins [50, 56, 57]. According to their base compositions, codons ending with C or G are avoided in all *Ophryotrocha* species, which is a trend observed for other annelid species [52].

Surprisingly, the gene order of the mitochondrial genome was not conserved as the positions of PCG and tRNA differed among the six *Ophryotrocha* species. Mitochondrial gene order tends to be highly conserved among metazoans [58] except in some groups such as molluscs [59, 60] and c [61]. Most frequent changes in gene order involve tRNA, while rearrangements between rRNAs or PCG are rarer [9]. Interestingly, we found rearrangements involving both tRNAs, PCG and ribosomal genes within congeneric species. Gene rearrangements have already been observed in other annelid families. Specimens from the Diurodrilidae [62], Chaetopteridae [12] and Syllidae [11] have shown different gene orders. However, previous findings suggest that these rearrangements are confined to basal lineages and should not be found in more recent lineages such as the Errantia and Sedentaria [12]. Our results do not provide support for this hypothesis, and are instead in line with findings by Ocerguera-Figueroa et al. [63], who reported gene rearrangement in more recent lineages. Several hypotheses have been proposed to explain these rearrangements: the role of tRNAs as mobile elements within the mitochondrial genome [55], intra-mitochondrial recombination [64], or the influence of the oxidative stress [65]. The latter hypothesis would explain why certain clades with species experiencing greater levels of oxidative stress are more prone to rearrangements compared to clades with species experiencing lower levels of oxidative stress [66]. As a consequence, it remains to be further tested whether annelid species with greater degrees of gene rearrangement are also those species that exhibit higher oxidative stress levels.

### Ribosomal genes on the minus strand

In addition, we report the presence of ribosomal genes on the minus strand. Usually, all annelid mitochondrial genes are encoded on the same strand except in the tubeworm *Owenia fusiformis*, the magelonid *Magelona mirabilis* (Johnston, 1865), and the ragworm *Laeonereis culveri* (Webster, 1879), which have one or two tRNA located on the minus strand [12, 67]. To explain this, it was hypothesised that, in the last common ancestor of annelids, all the genes were encoded on the same strand by chance wherein a ratchet effect took place, eliminating all the transcriptional elements and preventing the translocation of genes to the other strand [1, 68]. Weigert et al. [12] proposed two hypotheses to explain the translocation of genes between strands in annelids. The first hypothesis implies that in the last common ancestor of annelids, a single strand first encoded all genes and then underwent an inversion-transposition of the tRNAs and their transcriptional elements. The second hypothesis implies that the last common ancestor of annelids still had transcription signals on both strands and that these signals were kept in the basal lineages and lost in the more recent ones. However, *O. diadema* and *Laeonereis culveri* [67], two species found in most recent lineages, possess genes encoded on both strands, suggesting either (1) that a more recent ancestor still possessed transcript signals on both strands or (2) that tRNAs were transposed with transcript elements.

Indeed, sequencing strategies and/or annotation methodologies can influence the results in terms of length of the mitogenome sequence recovered or in the gene length [69]. Some studies have documented the presence of dissimilarities in the assembly and annotation of mitochondrial genomes that are not always associated with low coverage [69, 70]. In addition, not all the species are necessarily sensitive to this problem, which complicates the identification of a uniform approach to obtaining reliable mitochondrial genomes [69]. Since gene order is not affected by the methodology used, our results can be reasonably considered not to be an artefact.

### Phylogenetic relationships within the genus *Ophryotrocha* using mitogenomes and traditional marker

Based on our first mitogenome phylogeny of the genus *Ophryotrocha*, the two main clusters differed in their sexual strategies: one group including only gonochoric species (*O. japonica*, *O. labronica* and *O. robusta*), and the other including only hermaphroditic species (*O. adherens*, *O. diadema* and *O. puerilis*). A segregation based on the reproductive mode has been reported in previous phylogenetic studies [40, 41]. Genomic features differed between these two groups. The hermaphroditic species harboured negative AT-skew and GC-skew values in most of the PCG and ribosomal genes, as reported for other mitogenomes of annelids [50]. Gonochoric species had positive GC-Skew and negative AT-skew for each PCG. Codon usage was also different between the two-sexual modes: to code glutamic acid, the gonochoric species use the GAG codon, whereas the hermaphrodites use the GAA. Similar differences in codon usage remain to be confirmed in additional hermaphroditic and gonochoric *Ophryotrocha* species.

Based on the phylogeny of the *Ophryotrocha* genus we obtained, two main clades were identified: clade 1 that contains most of gonochoric species and clade 2 mostly composed of hermaphrodite species, but also including species with gonochoric strategies. Lending a closer look within the clade, reveal that a separation between reproductive modes was not as clear as it was reported in previous phylogenies including only a few species [40]. In addition, several groups were identified as *O. labronica*, this suggesting the existence of issues related to taxonomic identification. In contrast to Dahlgren et al. [40] and Heggoy et al. [41], we observed another clade within the hermaphrodite group. The separation of this clade was not linked to the reproductive mode of the species, although most of them are lacking information on their reproductive mode. Wilkund et al. [42] suggested a separation according to their habitat. As we do not possess this information for all species, it was not possible to comprehensively test for this hypothesis.

In addition, it is worth noticing the presence of different lineages with the same name in our phylogenies. This observation confirm the presence of cryptic species within groups that were originally described as independent species, as hypothesized for *O. labronica* lineages and for the *O. puerilis* complex, this confirming what reported in recent studies (e.g. [42, 43, 45]). In some cases, some gene sequences with different names corresponded to the same species, as for *O. obscura* and *O. sanya* (previously *O. vellae* by Paxton et al. [71]). In certain cases, the wrong taxonomic identification of the species may have occurred, prompting the incorrect association to a given reproductive mode, such as for sequences labeled as *O. diadema* #3 and *O. permanni* #2. Finally, we confirmed that *Iphitime* species do cluster in the *Ophryotrocha* genus [39, 41, 43, 45, 72, 73]. However, in our phylogeny, the *Iphitime* species are closely related to *O. adherens*, *O. puerilis* and *O. socialis*, whereas in the phylogeny from Heggoy et al. [40] the *Iphitime* specimen was closer to *O. gracilis* and *O. hartmanni*. Contrary to Taboada et al. [45], we found *O. clava* closely related to *O. jiaolongi* and the *Iphitime* species. This result was also observed by Zhang et al. [39].

As reported in previous studies, three different reproductive modes are present in the *Ophryotrocha* genus, i.e. gonochorism, simultaneous hermaphroditism and proterandrous hermaphroditism: the latter found in only one species, *O. puerilis* [31]. Contrary to other phyla, such as Mollusca and Arthrpoda, the Annelida phylum is known for its high diversity in reproductive modes, even within families [74]. The relative simplicity of their reproductive system and the relaxed morpho-physiological constraints to the evolution of alternative reproductive strategies seem to have favoured this remarkable variation of reproductive modes even among congeneric species [75]. Our results suggest that transitions between reproductive modes, i.e. gonochorism to hermaphroditism and vice versa, seem to have occurred multiple times along the evolutionary history of this genus. Indeed, both gonochoric and hermaphroditic species of the *Ophryotrocha* genus show some degree of sexual lability in the population that can potentially favour the expression of alternative reproductive strategies [76]. In particular, the presence of sexual phenotypes in the gonochoric species (i.e. pure male, male with a few oocytes, pure female, and female with a few sperm) is considered a vestigial trait of an ancestral hermaphroditic state [77]. Interestingly, our study shows that the ancestral reproductive strategy of this genus is most likely gonochorism, not hermaphroditism as previously reported [40, 41, 78]. Moreover, while clade 1 is composed of only gonochoristic species, clade 2 contained all modes of reproduction. These findings are in line with a recent study suggesting that transitions from gonochorism to hermaphroditism are more common than the reverse in many animal taxa. Factors promoting hermaphroditism in gonochoristic animals, such as low densities and inbreeding depression, are in fact more widespread or create stronger selection pressures than the conditions promoting gonochorism in hermaphroditic animals, such as high density and reproductive assurance [79].

The evolution of hermaphroditic strategies in this genus has been generally explained as an adaptation to conditions of low density, stabilized by poor mate search efficiency and high costs of searching [32]. In the Mediterranean Sea, the gonochoristic species *O. labronica* and *O. japonica* are indeed present in greater densities compared to the other hermaphrodite species [80]. In addition, Prevedelli et al. [31] demonstrated that gonochoristic and hermaphroditic species differed in a number of life-history traits. These differences confer to the former higher fitness and demographic advantages over the latter in conditions of high mate-search efficiency. However, the lack of information on environmental population densities and on life history for the majority of the *Ophryotrocha* species comprising our phylogeny prevents this hypothesis from being formally tested.

Finally, we cannot completely discard the idea that the ancestral reproductive mode could have been somewhat an “intermediate” one between gonochorism and hermaphroditism, given the documented wide variety of sexual phenotypes found in some species of these genus expressing these two extant forms of reproduction [76, 77]. Further research is therefore needed to better understand the phylogenetic relationship among *Ophryotrocha* species as emerging models for the investigation of evolutionary global change biology [37, 38].

## Conclusions

The descriptions of unique gene rearrangements within the *Ophryotrocha* genus are remarkable as they suggest that mitochondrial genomes in this taxonomic group are highly dynamic, signalling that gene rearrangements can occur more rapidly than previously thought. The within-genus PCG rearrangement refutes the idea that the gene order is conserved among the Errantia, although further studies are required to determine the mechanisms involved. The use of next generation sequencing techniques on *Ophryotrocha* has revealed the significant potential of these species as model organisms for studying evolutionary history within this genus. Moreover, this study displays the remarkable level of genetic diversification in annelids found even among closely related species. It highlights the need to increase the taxonomic representation in future phylogenetic studies for a more accurate understanding of this phylum’s diversity. Finally, developing an in-depth genomic understanding of the *Ophryotrocha* genus will help further the investigation of both evolution of life-history traits and the emerging field of evolutionary global change biology [37, 38, 81, 82].

## Methods

### Specimen collection, genomic extractions and sequencing

Specimens of the six *Ophryotrocha* species originated from individuals collected in 2008 (*O. robusta*, *O. diadema*, *O. adherens*) in the harbour of Porto Empedocle (Italy; 37°17′4″N, 13°31′3″E) and in 2015 (*O. labronica*, *O. japonica, O. puerilis*) in the harbour of La Spezia (Italy, 44° 6′ 24″ N, 9° 49′ 45″ E). Species identification was performed using morphological and reproductive traits as described by Simonini et al. [80]. For each species, three samples (each from a single breeding pair) of 30 individuals were used for mtDNA extraction. Genomic extractions were performed using QIAGEN’s DNeasy Blood and Tissue Kit with RNAse according to the manufacturer’s protocol. Quantification of DNA was done with Quant-iT™ PicoGreen® dsDNA Assay Kit (Invitrogen™, Canada following the manufacter’s protocol. Genomic DNA (500 ng) was mechanically fragmented for 40 s. using a covaris M220 (Covaris, Woburn MA, USA) with default settings. Fragmented DNA was transferred to PCR tubes and library synthesis was performed with the NEB Next Ultra II (New England Biolabs) according to manufacturer’s instructions. TruSeq HT adapters (Illumina, SanDiego, CA, USA) were used to barcode the samples. The libraries were quantified and pooled using an equimolar ratio and sequenced on an Illumina MiSeq 300 base-pair (bp) paired-end run (600 cycle, v3 kit) at the Plateforme d’Analyses Génomiques of the Institut de Biologie Intégrative et des Systèmes (Laval University, Quebec, Canada).

### Assembly and annotations

The quality of the sequencing was assessed with Fastqc [83], and adapters were removed with Trimmomatic [84] available in usegalaxy.org [85]. Default parameters were used to retrieve mitochondrial genomes using the perl script Novoplasty2.7.2 [86]. Briefly, based on a seed-and-extend algorithm, mitochondrial genome is retrieved from the whole genome sequencing data, using a related or distant single seed sequence [87]. For each species, we used a fragment of COX1 as seed: JQ310756.1 for *O. adherens*, JQ310758.1 for *O. diadema*, EF46454.1 for *O. japonica*, KF305814.1 for *O. labronica*, EF 464544.1 for *O. puerilis*, EF464547.1 for *O. robusta*. As no mitochondrial genome from close relatives of each species was available, no references were used for the assembly. Annotation was performed with MITOS2 Web Server [87], verified with ORF finder [88] and ARWEN [89], and visualized using GeSeq [90]. Determination of the A + T content of protein-coding genes, tRNA genes, rRNA genes and the RSCU was performed with DAMBE 6 [91]. All the mitochondrion genomes have been deposited in Genbank under the following accession number: MT737360 (*O. robusta*), MT737361 (*O. labronica*), MT737362 (*O. japonica*), MT737363 (*O. adherence*), MT737364 (*O. diadema*), MT737365 (*O. puerilis*).

### Gene rearrangement

CREx [92] was used to examine the possible scenarios of rearrangement between pairs of complete genomes. Briefly, this method compares two genomes and determines the most parsimonious scenario that has led to the observed rearrangement accounting for duplication reversals, transpositions or other events. As the tRNA order is not conserved among Annelida species [12], we kept only the PCG order to infer the possible scenarios of rearrangement. We examined all the rearrangement scenarios among *Ophryotrocha* species and other gene orders known for annelids obtained from Lavrov and Lang [93], Mwinyi et al. [54] and Weigert et al. [12]. Results were visualized with the R-package genoPlotR [94].

### Phylogeny reconstruction

Phylogenies were assessed using maximum likelihood and Bayesian inference. First, PCG from whole mitochondrial sequences were used to reconstruct phylogenies for the six *Ophryotrocha* species considered. Each PCG was separately aligned and then concatenated using seaview4 [95]. Poorly aligned regions were removed using Gblocks [96] with default parameters. This step was performed on both nucleotide and amino-acid sequences of PCG. Two species were used as outgroup in the phylogenies: *Marphysa sanguinea* (NC_023124.1) and *O. fusiformis* (NC_028712.1). Secondly, in order to investigate the relationship among *Ophryotrocha* species, we built a phylogeny based on two mitochondrial fragments, the COI gene and the 16S, and a nuclear fragment from Histone 3 (H3). First, we retrieved the Histone 3 fragment for each *Ophryotrocha* species used in this study using a BLASTn search available in Galaxy [85] with a H3 fragment from a close relative available in Genbank. Sequences of H3 have been deposited in Genbank (MT733538-MT733543). In order to determine if the three genes could be concatenated for phylogenetic analyses, we tested the congruence among the three distance matrices genes using the congruence among distance matrices approach (CADM) developed by [97, 98] available in ape R-package [99]. Briefly, the CADM tests for the presence of incongruency among all the distance matrices. The significance of the test was performed with a 1000 permutations. All three distance matrices were obtained in MEGA5 [100]. No incongruence was detected among the matrices (W = 0.65, *p* = 0.001) and all the sequences were concatenated. A total of 71 sequences representing 46 species from Genbank (listed in Additional file 16) and the six-species used in this study were aligned for each gene with MAFFT version 7 [101] available at https://mafft.cbrc.jp/alignment/software . All the COXI aligned sequences were trimmed to 400 bp length and all the H3 sequences were trimmed to 298 bp length. All three genes were concatenated. We used a combination of close and distant species as outgroups: *Eunice pennata*, *Eunice norvegica*, *Protodorvillea gracilis*, *Dorvillea erucaeformis*. For each phylogeny, the most adapted evolutionary model was determined based on Bayesian information criterion as implemented in W-IQ TREE [102]. Maximum likelihood (ML) trees were subsequently generated using IQ-Tree [103] and branch support estimated using the Shimodaira-Hasegawa approximate likelihood ratio test (aLTR), as described in Anisimova and Gascuel [104]. For each dataset, Bayesian inferences (BI) were performed on two runs until convergence was reached under the appropriate evolutionary model. Tree sampling was done every 1000 generations with a burn-in of 25%. Bayesian reconstructions were performed using MrBayes 3.2.6 [105] available on the CIPRES gateway [106]. The posterior probabilities (pp) were obtained for the 50% majority-rule consensus tree. Strong support of branches was considered when pp. ≥ 0.95, whereas intermediate support was defined with pp. values between 0.85 and 0.94.

### Ancestral state reconstruction of reproductive trait in *Ophryotrocha*

Information about the reproductive mode of each species (gonochoric, simultaneous hermaphrodite and protandrous hermaphrodite) was retrieved from the literature (Additional file 16) and all species coded accordingly. When no information on the reproductive mode was found, species were marked as unknown. Parsimony ancestral trait reconstruction was performed on both ML and BI phylogeny in order to find the ancestral states that minimize the number of changes according to the phylogeny. Analysis was conducted in Mesquite v3.4 [107].

In addition to the maximum parsimony reconstruction, we also used a method based on Bayesian MCMC sampling methods to reconstruct the ancestral reproductive mode of the genus *Ophryotrocha* as implemented in BEAST2 [108]. In particular, we used the Bayesian phylogeny previously obtained and estimate the posterior probability of the state for each ancestor for each node of the tree. We conducted 10,000,000 iterations and sampled parameters every 10,000 generations. The posterior distribution was first verified with Tracer1.7 [109]. We discarded the first 25% samples of states obtained as burn-in as implemented in TreeAnnotaor from the BEAST2 package.

## Supplementary Information

**Additional file 1. **Main literature on the genus *Ophryotrocha*.

**Additional file 2. **Genome annotation for *Ophryotrocha adherens*.

**Additional file 3. **Genome annotation for *Ophryotrocha diadema*.

**Additional file 4. **Genome annotation of *Ophryotrocha japonica.*

**Additional file 5. **Genome annotation of *Ophryotrocha labronica.*

**Additional file 6. **Genome annotation of *Ophryotrocha puerilis.*

**Additional file 7. **Genome annotation of *Ophryotrocha robusta.*

**Additional file 8. **tRNA structures in *O. adherens*.

**Additional file 9. **tRNA structures in *O. diadema.*

**Additional file 10. **tRNA structures in *O. japonica.*

**Additional file 11. **tRNA structures in *O. labronica.*

**Additional file 12. **tRNA structures in *O. puerilis.*

**Additional file 13. **tRNA structures in *O. robusta.*

**Additional file 14. **Matrix of the comparison between gene order of the six *Ophryotrocha* species investigated.

**Additional file 15. **Matrix of the comparison between gene order of *Ophryotrocha* and other annelids.

**Additional file 16. **List of *Ophryotrocha* species used for the phylogeny.

**Additional file 17.** Ancestral reproductive mode reconstruction based on Bayesian posterior probabilities.

## Data Availability

All mitochondrial sequences and the Histone 3 fragment were deposited under the accession number MT737360-MT737365 and MT733538-MT733543 in the Genbank database. Alignments and script were available as Supplementary file. The accession number of all sequences of COI, 16S and H3 used in this study are listed in the additional file 16 of the Supplementary Material and are available in Genbank database. Mitogenome of *Marphysa sanguinea* (NC_023124.1) and *O. fusiformis* (NC_028712.1), used for phylogenies reconstruction, are available in Genbank. The following accession numbers: JQ310756.1, JQ310758.1, KF305814.1, EF 464544.1 and EF464547.1 are available in Genbank.

## Abbreviations

mt: Mitochondrial
PCG: Protein Coding Gene
COI: Cytochrome oxidase I
RSCU: Relative Synonymous Codon Usage
BI: Bayesian inference
ML: Maximum likelihood
aLTR: Approximate likelihood ratio test
pp: Posterior probabilities
