# Supplementary material for: Extensive gene rearrangements in the mitogenomes of congeneric annelid species and insights on the evolutionary history of the genus Ophryotrocha
Source: BMC Genomics. 2020 Nov 23;21:815. doi: 10.1186/s12864-020-07176-8 (PMC7682095; doi:10.1186/s12864-020-07176-8)
Supplement: Supplementary file 1 — Additional file 1. Main literature on the genus Ophryotrocha. [file 12864_2020_7176_MOESM1_ESM.docx]

**Additional file 1.** Main literature on the genus *Ophryotrocha*.

| **Biology sub-disciplines** | **Main references** |
| --- | --- |
| Comparative biology | Macnaughton et al., 2010, 2011; Müller et al., 2002 |
|  | |
| Cytology and Histology | Berruti et al., 1978; Emanuelsson, 1985; Emanuelsson et al., 1985; Grothe et al., 1986, 1987; Huth, 1934; Jacobsohn, 2003; Murra et al., 2012; Neuman, 1983; Pfannenstiel et al., 1990; Robotti et al., 1991; Schlawny et al., 1991a; Sella, 1995; Sunner et al., 1971; Vitturi et al., 2000 |
|  |  |
|  |  |
| Developmental biology | Bergter et al., 2008; Brubacher, 2010; Brubacher et al., 2009, 2011; Emanuelsson, 1969, 1973, 1974, 1992; Emanuelsson et al., 1978, 1985; Franchini, 2008; Heby et al., 1978, 1981; Pfannenstiel, 1984; Pfannenstiel et al., 1982a,b |
|  |  |
| Ecology (Experimental and field ecology, macroecology community ecology) | Åkesson, 1972b, 1976b, 1978, 1982; Åkesson et al., 1978, 2005; Bacci et al., 1953, 1967; Berruti et al., 1980; Cassai et al., 1999; Delgado et al., 2003; Hilbig, 2004; Kegel et al., 1983; Knowles et al., 1994, 1997; Levin et al., 2006, 2013; Levinton, 1983; Levinton et al., 1983; Marchionni et al., 1981; Massamba-N’Siala et al., 2011, 2012, 2014; Mercier et al., 2014; Minetti et al., 2013; Pfannenstiel, 1974; Premoli et al., 1995; Prevedelli et al., 1998, 2001, 2002, 2003a,b, 2005, 2006; Salvo et al., 2015a, 2018; Sella, 1985; Sella et al., 1983, 1993b; Simonini et al., 2003, 2009, 2010; Thornhill et al., 2009; Toboada et al., 2016; Verkaik et al., 2017; Wilson, 1991 |
|  |  |
|  |  |
|  |  |
|  |  |
| Ecotoxicology | Åkesson, 1970; Bao-ling, 1981; Brown et al., 1971; Carr et al., 1977; Fang et al., 2018; Hooftman et al 1980; Jernelöv et al., 1972; Klöckner, 1979; Lance et al., 2012; Parker, 1984; Pocklington et al., 1992; Qie et al., 2017; Qiu et al., 2005; Reish et al., 1978; Røed, 1980; Rosenberg et al., 1975; Saliba et al., 1973 |
|  |  |
|  |  |
| Environmental biology | Gibbs, 1969; Lacroix et al., 2005; Lin et al., 2008; Mair et al., 1987; Pereira et al., 2004; Salvo et al., 2015b, 2017; Wildish et al., 2005 |
|  |  |
| Evolutionary ecology (Evolution of sex strategies) | Anthes, 2010; Bacci, 1965; Baeza, 2007; Berglund, 1986, 1990, 1991; Cannarsa et al., 2017; Di Bona et al, 2010, 2015; Lorenzi et al., 2000, 2006, 2008, 2014a,b, 2015, 2018; Meconcelli et al., 2015, 2017; Picchi et al., 2018; Santi et al., 2018; Schleicherova et al., 2005, 2006, 2010, 2013, 2014; Sella, 1988, 1990, 1991; Sella et al., 1992, 1997, 2000, 2003 |
|  |  |
|  |  |
| Genetics, Systematics and Taxonomy | Cannarsa et al., 2016; Costa-Paiva et al., 2017; Dahlgren et al., 2001; Cossu et al., 2015; Emanuelsson, 1971; Gambi et al., 1997; Heggøy et al., 2007; Lattig et al., 2015, 2016; Pleije et al., 1996; Ravara et al., 2015; Satheeshkumar et al., 2010; Sella et al., 1993b; Tilic et al., 2016; Wiklund et al., 2012 |
|  |  |
|  |  |
|  |  |
| Global change biology | Chakravarti et al., 2016; Gibbin et al., 2017a,b; Jarrold et al., 2019; Rodríguez‐Romero et al., 2016 |
|  |  |
| Immunology | Franchini and Ottaviani, 2007; Schlawny et al., 1991b |
|  |  |
| Palaeontology | Caron et al., 2007; Eriksson et al., 2000 |
|  |  |
| Zoology and Taxonomy (Morphology, species identification) | Åkesson, 1972a, 1974a,b, 1976a; del Carmen Brito et al., 2003; Hilbig et al., 1991; Josefson, 1975; Martin et al., 1991; Miura, 1997; Murray, 2016; Nunez et al., 2014; Ockelmann et al., 1990; Oug, 1990; Paavo et al., 2000; Paxton, 2004; Paxton et al., 2007, 2009, 2010a,b, 2011; Pfannenstiel et al., 1982c; Rhode, 1989, 1990; Salvo et al, 2014; Schlawny et al., 1991c; Sicinski et al., 1993; Starunov, 2019; Toboada et al., 2013, 2017; Troyer et al., 1979; Wiklund et al., 2009; Zavarzina et al., 1991; Zhang et al., 2017 |
|  |  |
|  |  |
