# Supplementary material for: Extensive gene rearrangements in the mitogenomes of congeneric annelid species and insights on the evolutionary history of the genus Ophryotrocha
Source: BMC Genomics. 2020 Nov 23;21:815. doi: 10.1186/s12864-020-07176-8 (PMC7682095; doi:10.1186/s12864-020-07176-8)
Supplement: Supplementary file 2 — Additional file 2. Genome annotation for Ophryotrocha adherens. [file 12864_2020_7176_MOESM2_ESM.docx]

**Additional file 2.** Genome annotation for *Ophryotrocha adherens*.

| ***Ophryotrocha adherens*** | | | | | | | |
| --- | --- | --- | --- | --- | --- | --- | --- |
| **Name** | **Start** | **Stop** | **Strand** | **Length** | **ovl/nc** | **Codons** | **Anticodon** |
| Non coding region | 1 | 213 | + | 212 | 14 |  |  |
| tRNA-Asp | 227 | 290 | + | 63 | 15 |  | GTC |
| atp8 | 305 | 478 | + | 173 | 15 | ATG/TAA |  |
| tRNA-Pro | 493 | 558 | + | 65 | 12 |  | TGG |
| tRNA-Arg | 570 | 633 | + | 63 | 48 |  | TCG |
| rrnL | 681 | 1778 | + | 1097 | 1 |  |  |
| tRNA-Trp | 1779 | 1842 | + | 63 | 2 |  | TCA |
| tRNA-Leu1 | 1844 | 1907 | + | 63 | -50 |  | TAA |
| nad1 | 1857 | 2837 | + | 980 | 3 | ATT/TAA |  |
| tRNA-Lys | 2840 | 2902 | + | 62 | -1 |  | TTT |
| tRNA-Ile | 2901 | 2965 | + | 64 | 1 |  | GAT |
| nad3 | 2966 | 3313 | + | 347 | -1 | ATG/TAA |  |
| tRNA-Ser1 | 3312 | 3371 | + | 59 | 15 |  | TCT |
| nad2 | 3386 | 4357 | + | 971 | 0 | ATG/TAA |  |
| cox1 | 4357 | 5889 | + | 1532 | 3 | ATG/TAA |  |
| tRNA-Asn | 5892 | 5954 | + | 62 | 2 |  | GTT |
| cox2 | 5956 | 6639 | + | 683 | -9 | ATG/TAA |  |
| cox3 | 6630 | 7424 | + | 794 | -99 | ATT/TAA |  |
| cytb | 7325 | 8710 | + | 1385 | -12 | ATT/TAA |  |
| nad6 | 8698 | 9210 | + | 512 | 4 | ATG/TAA |  |
| atp6 | 9214 | 9933 | + | 719 | -19 | ATT/TAA |  |
| tRNA-His | 9914 | 9978 | + | 64 | -2 |  | GTG |
| nad5 | 9976 | 11727 | + | 1751 | 5 | ATT/TAA |  |
| tRNA-Glu | 11732 | 11796 | + | 64 | 0 |  | TTC |
| tRNA-Thr | 11796 | 11859 | + | 63 | 1 |  | TGT |
| nad4l | 11860 | 12150 | + | 290 | -6 | ATG/TAA |  |
| nad4 | 12144 | 13484 | + | 1340 | -1 | ATG/TAA |  |
| tRNA-Ser2 | 13483 | 13547 | + | 64 | 0 |  | TGA |
| tRNA-Gly | 13547 | 13609 | + | 62 | 21 |  | TCC |
| rrnS | 13630 | 14423 | + | 793 | -7 |  |  |
| tRNA-Tyr | 14416 | 14477 | + | 61 | 1 |  | GTA |
| tRNA-Ala | 14478 | 14540 | + | 62 | 2 |  | TGC |
| tRNA-Met | 14542 | 14608 | + | 66 | 1 |  | CAT |
| tRNA-Cys | 14609 | 14672 | + | 63 | 2 |  | GCA |
| tRNA-Leu2 | 14674 | 14736 | + | 62 |  |  | TAG |

ovl= overlapping region, nc= non-coding region
