# Supplementary material for: Extensive gene rearrangements in the mitogenomes of congeneric annelid species and insights on the evolutionary history of the genus Ophryotrocha
Source: BMC Genomics. 2020 Nov 23;21:815. doi: 10.1186/s12864-020-07176-8 (PMC7682095; doi:10.1186/s12864-020-07176-8)
Supplement: Supplementary file 3 — Additional file 3. Genome annotation for Ophryotrocha diadema. [file 12864_2020_7176_MOESM3_ESM.docx]

**Additional file 3.** Genome annotation for *Ophryotrocha diadema*.

| ***Ophryotrocha diadema*** | | | | | | | |
| --- | --- | --- | --- | --- | --- | --- | --- |
| **Name** | **Start** | **Stop** | **Strand** | **Length** | **ovl/nc** | **Codons** | **Anticodon** |
| tRNA-Ser | 76 | 140 | + | 64 | 3 |  | TCT |
| cox1 | 143 | 1759 | + | 1616 | -1 | TTG/TAA |  |
| tRNA-Tyr | 1758 | 1820 | + | 62 | -34 |  | GTA |
| nad1 | 1786 | 2754 | + | 968 | -1 | ATT/TAG |  |
| tRNA-Ile | 2753 | 2815 | + | 62 | -26 |  | GAT |
| nad3 | 2789 | 3163 | + | 374 | 10 | TTG/TAG |  |
| nad2 | 3173 | 4081 | + | 908 | 23 | ATG/TAA |  |
| cox2 | 4104 | 4796 | + | 692 | -1 | ATT/TAA |  |
| tRNA-Trp | 4795 | 4859 | + | 64 | 6 |  | TCA |
| tRNA-Asp | 4865 | 4926 | + | 61 | 1 |  | GTC |
| atp8 | 4927 | 5091 | + | 164 | -9 | ATG/TAA |  |
| cox3 | 5082 | 5876 | + | 794 | 15 | ATG/TAG |  |
| tRNA-Asn | 5891 | 5954 | + | 63 | 2 |  | GTT |
| tRNA-Lys | 5956 | 6017 | + | 61 | 2 |  | TTT |
| tRNA-Gln | 6019 | 6085 | + | 66 | 1 |  | TTG |
| nad6 | 6086 | 6541 | + | 455 | 6 | ATG/TAG |  |
| cytb | 6547 | 7686 | + | 1139 | -3 | ATG/TAA |  |
| atp6 | 7683 | 8405 | + | 722 | 0 | ATA/TAA |  |
| tRNA-His | 8405 | 8467 | + | 62 | 1 |  | GTG |
| tRNA-Arg | 8468 | 8522 | + | 54 | 1 |  | TCG |
| nad5 | 8523 | 10232 | + | 1709 | -19 | ATG/TAA |  |
| tRNA-Phe | 10213 | 10273 | + | 60 | 0 |  | GAA |
| tRNA-Glu | 10273 | 10334 | + | 61 | -4 |  | TTC |
| tRNA-Pro | 10330 | 10392 | + | 62 | 0 |  | YGG |
| tRNA-Thr | 10392 | 10452 | + | 60 | -36 |  | TGT |
| nad4l | 10416 | 10742 | + | 326 | -24 | TTG/TAA |  |
| nad4 | 10718 | 12076 | + | 1358 | 0 | ATC/TAA |  |
| tRNA-Ser | 12076 | 12138 | + | 62 | 1 |  | TGA |
| tRNA-Ala | 12139 | 12200 | + | 61 | -1 |  | TGC |
| tRNA-Val | 12199 | 12258 | + | 59 | 177 |  | TAC |
| tRNA-Cys | 12435 | 12495 | + | 60 | 58 |  | GCA |
| tRNA-Gly | 12553 | 12614 | - | 61 | 4 |  | TCC |
| tRNA-Leu | 12618 | 12682 | - | 64 | -10 |  | TAG |
| rrnS | 12672 | 13467 | - | 795 | -1 |  |  |
| tRNA-Met | 13466 | 13529 | - | 63 | 1 |  | CAT |
| tRNA-Leu | 13530 | 13590 | - | 60 | -18 |  | TAA |
| rrnL | 13572 | 14673 | - | 1101 | 1 |  |  |
| Non coding region | 14674 | 75 | + | 1197 |  |  |  |

ovl= overlapping region, nc= non-coding region.
