# Supplementary material for: Extensive gene rearrangements in the mitogenomes of congeneric annelid species and insights on the evolutionary history of the genus Ophryotrocha
Source: BMC Genomics. 2020 Nov 23;21:815. doi: 10.1186/s12864-020-07176-8 (PMC7682095; doi:10.1186/s12864-020-07176-8)
Supplement: Supplementary file 4 — Additional file 4. Genome annotation of Ophryotrocha japonica. [file 12864_2020_7176_MOESM4_ESM.docx]

**Additional file 4.** Genome annotation for *Ophryotrocha japonica*.

| ***Ophryotrocha japonica*** | | | | | | | |
| --- | --- | --- | --- | --- | --- | --- | --- |
| **Name** | **Start** | **Stop** | **Strand** | **Length** | **ovl/nc** | **Codons** | **Anticodon** |
| tRNA-Phe | 1 | 60 | + | 59 | 7 |  | GAA |
| tRNA-Thr | 67 | 130 | + | 63 | 4 |  | TGT |
| cox2 | 134 | 877 | + | 743 | 8 | ATG/TAG |  |
| tRNA-Trp | 885 | 948 | + | 63 | 4 |  | TCA |
| atp8 | 952 | 1113 | + | 161 | 1 | ATG/TAA |  |
| cox3 | 1114 | 1896 | + | 782 | 3 | ATG/TAA |  |
| tRNA-Gln | 1899 | 1965 | + | 66 | 1 |  | TTG |
| nad6 | 1966 | 2412 | + | 446 | 4 | ATG/TAA |  |
| cytb | 2416 | 3552 | + | 1136 | 14 | GTG/TAG |  |
| atp6 | 3566 | 4258 | + | 692 | 16 | ATG/TAG |  |
| tRNA-Arg | 4274 | 4334 | + | 60 | 2 |  | TCG |
| tRNA-His | 4336 | 4401 | + | 65 | 1 |  | GTG |
| nad5 | 4402 | 6084 | + | 1682 | 0 | ATG/TAA |  |
| tRNA-Ser1 | 6084 | 6142 | + | 58 | 2 |  | TCT |
| nad2 | 6144 | 7100 | + | 956 | 34 | ATG/TAG |  |
| cox1 | 7134 | 8660 | + | 1526 | 11 | ATT/TAA |  |
| tRNA-Cys | 8671 | 8733 | + | 62 | 1 |  | GCA |
| tRNA-Asn | 8734 | 8798 | + | 64 | 17 |  | GTT |
| nad4l | 8815 | 9099 | + | 284 | -6 | ATG/TAA |  |
| nad4 | 9093 | 10415 | + | 1322 | 8 | ATG/TAA |  |
| tRNA-Ser2 | 10423 | 10481 | + | 58 | 1 |  | TGA |
| tRNA-Ala | 10482 | 10541 | + | 59 | -1 |  | TGC |
| tRNA-Met | 10540 | 10604 | + | 64 | -2 |  | CAT |
| rrnS | 10602 | 11337 | + | 735 | 4 |  |  |
| tRNA-Gly | 11341 | 11404 | + | 63 | 3 |  | TCC |
| tRNA-Val | 11407 | 11461 | + | 54 | 1 |  | TAC |
| rrnL | 11462 | 12534 | + | 1072 | 1 |  |  |
| tRNA-Tyr | 12535 | 12595 | + | 60 | 13 |  | GTA |
| tRNA-Leu1 | 12608 | 12669 | + | 61 | -23 |  | TAG |
| nad1 | 12646 | 13581 | + | 935 | 3 | ATG/TAA |  |
| tRNA-Lys | 13584 | 13647 | + | 63 | -1 |  | TTT |
| tRNA-Ile | 13646 | 13707 | + | 61 | -26 |  | GAT |
| nad3 | 13681 | 14055 | + | 374 | 99 | GTG/TAG |  |
| tRNA-Glu | 14154 | 14218 | + | 64 | 4 |  | TTC |
| tRNA-Pro | 14222 | 14287 | + | 65 | 26 |  | TGG |
| tRNA-Leu2 | 14313 | 14376 | + | 63 | 1 |  | TAA |
| Non coding region | 14377 | 14710 | + | 333 |  |  |  |

ovl= overlapping region, nc=non-coding region
