# Supplementary material for: Extensive gene rearrangements in the mitogenomes of congeneric annelid species and insights on the evolutionary history of the genus Ophryotrocha
Source: BMC Genomics. 2020 Nov 23;21:815. doi: 10.1186/s12864-020-07176-8 (PMC7682095; doi:10.1186/s12864-020-07176-8)
Supplement: Supplementary file 5 — Additional file 5. Genome annotation of Ophryotrocha labronica. [file 12864_2020_7176_MOESM5_ESM.docx]

**Additional file 5.** Genome annotation for *Ophryotrocha labronica*.

| ***Ophryotrocha labronica*** | | | | | | | |
| --- | --- | --- | --- | --- | --- | --- | --- |
| **Name** | **Start** | **Stop** | **Strand** | **Length** | **ovl/nc** | **Codons** | **Anticodon** |
| tRNA-Phe | 413 | 475 | + | 62 | 5 |  | GAA |
| tRNA-Thr | 480 | 545 | + | 65 | -2 |  | TGT |
| cox2 | 543 | 1286 | + | 743 | -1 | GTG/TAA |  |
| tRNA-Trp | 1285 | 1350 | + | 65 | 4 |  | TCA |
| atp8 | 1354 | 1512 | + | 158 | 1 | ATG/TAA |  |
| cox3 | 1513 | 2295 | + | 782 | 8 | ATG/TAA |  |
| tRNA-Gln | 2303 | 2366 | + | 63 | 1 |  | TTG |
| nad6 | 2367 | 2816 | + | 449 | 3 | ATG/TAG |  |
| cytb | 2819 | 3949 | + | 1130 | 11 | GTG/TAA |  |
| atp6 | 3960 | 4655 | + | 695 | 3 | ATG/TAG |  |
| tRNA-Arg | 4658 | 4717 | + | 59 | 1 |  | TCG |
| tRNA-His | 4718 | 4780 | + | 62 | 1 |  | GTG |
| nad5 | 4781 | 6448 | + | 1667 | -1 | ATG/TAA |  |
| tRNA-Ser1 | 6447 | 6504 | + | 57 | 2 |  | TCT |
| nad2 | 6506 | 7471 | + | 965 | -42 | ATG/TAG |  |
| cox1 | 7429 | 9024 | + | 1595 | 11 | ATT/TAA |  |
| tRNA-Cys | 9035 | 9093 | + | 58 | 1 |  | GCA |
| tRNA-Asn | 9094 | 9155 | + | 61 | -5 |  | GTT |
| nad4l | 9150 | 9455 | + | 305 | -6 | ATT/TAA |  |
| nad4 | 9449 | 10771 | + | 1322 | -1 | GTG/TAA |  |
| tRNA-Ser2 | 10770 | 10827 | + | 57 | 1 |  | TGA |
| tRNA-Ala | 10828 | 10890 | + | 62 | -1 |  | TGC |
| tRNA-Met | 10889 | 10952 | + | 63 | -2 |  | CAT |
| rrnS | 10950 | 11697 | + | 747 | -4 |  |  |
| tRNA-Gly | 11693 | 11754 | + | 61 | 4 |  | TCC |
| tRNA-Val | 11758 | 11813 | + | 55 | 143 |  | TAC |
| tRNA-Pro? | 11956 | 12014 | + | 58 | -200 |  | AC? |
| rrnL | 11814 | 12877 | + | 1063 | 1 |  |  |
| tRNA-Tyr | 12878 | 12937 | + | 59 | 0 |  | GTA |
| tRNA-Leu1 | 12937 | 12997 | + | 60 | 6 |  | TAG |
| tRNA-Leu2 | 13003 | 13067 | + | 64 | -41 |  | TAA |
| nad1 | 13026 | 13979 | + | 953 | 3 | ATT/TAA |  |
| tRNA-Lys | 13982 | 14046 | + | 64 | -1 |  | TTT |
| tRNA-Ile | 14045 | 14106 | + | 61 | -26 |  | GAT |
| nad3 | 14080 | 14454 | + | 374 | 4 | ATG/TAG |  |
| tRNA-Glu | 14458 | 14521 | + | 63 | 1 |  | TTC |
| tRNA-Asp | 14522 | 14582 | + | 60 | 0 |  | GTC |
| Non coding region | 14582 | 15981 | + | 1399 |  |  |  |

ovl= overlapping region, nc=non-coding region
