# Supplementary material for: Extensive gene rearrangements in the mitogenomes of congeneric annelid species and insights on the evolutionary history of the genus Ophryotrocha
Source: BMC Genomics. 2020 Nov 23;21:815. doi: 10.1186/s12864-020-07176-8 (PMC7682095; doi:10.1186/s12864-020-07176-8)
Supplement: Supplementary file 6 — Additional file 6. Genome annotation of Ophryotrocha puerilis. [file 12864_2020_7176_MOESM6_ESM.docx]

**Additional file 6.** Genome annotation of *Ophryotrocha puerilis*.

| ***Ophryotrocha puerlis*** | | | | | | | |
| --- | --- | --- | --- | --- | --- | --- | --- |
| **Name** | **Start** | **Stop** | **Strand** | **Length** | **ovl/nc** | **Codons** | **Anticodon** |
| tRNA-Glu | 185 | 246 | + | 61 | -47 |  | TTC |
| rrnL | 199 | 1338 | + | 1139 | -27 |  |  |
| tRNA-Tyr | 1311 | 1370 | + | 59 | 1 |  | GTA |
| tRNA-Ser | 1371 | 1431 | + | 60 | 1 |  | TCT |
| nad2 | 1432 | 2403 | + | 971 | 0 | ATG/TAA |  |
| tRNA-Leu | 2403 | 2465 | + | 62 | -20 |  | TAA |
| nad1 | 2445 | 3398 | + | 953 | -1 | ATG/TAA |  |
| tRNA-Trp | 3397 | 3460 | + | 63 | 1 |  | TCA |
| tRNA-Lys | 3461 | 3526 | + | 65 | 0 |  | TTT |
| tRNA-Ile | 3526 | 3587 | + | 61 | 1 |  | GAT |
| nad3 | 3588 | 3971 | + | 383 | -37 | ATG/TAA |  |
| tRNA-Asn | 3934 | 3995 | + | 61 | 16 |  | GTT |
| cox1 | 4011 | 5546 | + | 1535 | 8 | GTG/TAA |  |
| cox2 | 5554 | 6243 | + | 689 | -1 | ATA/TAA |  |
| tRNA-Asp | 6242 | 6302 | + | 60 | 4 |  | GTC |
| atp8 | 6306 | 6461 | + | 155 | 7 | ATG/TAA |  |
| cox3 | 6468 | 7250 | + | 782 | 3 | ATG/TAA |  |
| tRNA-Gln | 7253 | 7319 | + | 66 | -23 |  | TTG |
| nad6 | 7296 | 7769 | + | 473 | 2 | ATA/TAA |  |
| cytb | 7771 | 8910 | + | 1139 | 1 | GTG/TAA |  |
| atp6 | 8911 | 9642 | + | 731 | -38 | ATC/TAA |  |
| tRNA-Arg | 9604 | 9667 | + | 63 | 0 |  | TCG |
| tRNA-His | 9667 | 9727 | + | 60 | 1 |  | GTG |
| nad5 | 9728 | 11413 | + | 1685 | 1 | ATG/TAG |  |
| tRNA-Thr | 11414 | 11475 | + | 61 | 2 |  | TGT |
| tRNA-Phe | 11477 | 11536 | + | 59 | 3 |  | GAA |
| tRNA-Pro | 11539 | 11604 | + | 65 | -8 |  | TGG |
| nad4l | 11596 | 11895 | + | 299 | -24 | ATT/TAG |  |
| nad4 | 11871 | 13232 | + | 1361 | -1 | ATC/TAA |  |
| tRNA-Ser | 13231 | 13291 | + | 60 | 2 |  | TGA |
| tRNA-Ala | 13293 | 13354 | + | 61 | -1 |  | TGC |
| tRNA-Met | 13353 | 13415 | + | 62 | -1 |  | CAT |
| rrnS | 13414 | 14202 | + | 788 | -7 |  |  |
| tRNA-Leu | 14195 | 14255 | + | 60 | 0 |  | TAG |
| tRNA-Gly | 14255 | 14310 | + | 55 | 3 |  | TCC |
| tRNA-Cys | 14313 | 14373 | + | 60 | 1 |  | GCA |
| Non coding region | 14374 | 184 | + | 1751 |  |  |  |

ovl= overlapping region, nc= non-coding region
