# Supplementary material for: Extensive gene rearrangements in the mitogenomes of congeneric annelid species and insights on the evolutionary history of the genus Ophryotrocha
Source: BMC Genomics. 2020 Nov 23;21:815. doi: 10.1186/s12864-020-07176-8 (PMC7682095; doi:10.1186/s12864-020-07176-8)
Supplement: Supplementary file 7 — Additional file 7. Genome annotation of Ophryotrocha robusta. [file 12864_2020_7176_MOESM7_ESM.docx]

**Additional file 7.** Genome annotation of *Ophryotrocha robusta*.

| ***Ophryotrocha robusta*** | | | | | | | |
| --- | --- | --- | --- | --- | --- | --- | --- |
| **Name** | **Start** | **Stop** | **Strand** | **Length** | **ovl/nc** | **Codons** | **Anticodon** |
| cox2 | 158 | 865 | + | 707 | 4 | ATA/TAA |  |
| tRNA-Trp | 869 | 930 | + | 61 | 0 |  | TCA |
| tRNA-Asp | 930 | 990 | + | 60 | 2 |  | GTC |
| atp8 | 992 | 1150 | + | 158 | -6 | ATG/TAG |  |
| cox3 | 1144 | 1932 | + | 788 | 1 | TTG/TAA |  |
| tRNA-Gln | 1933 | 1997 | + | 64 | 1 |  | TTG |
| nad6 | 1998 | 2438 | + | 440 | 0 | ATG/TAA |  |
| cytb | 2438 | 3568 | + | 1130 | -7 | ATG/TAA |  |
| atp6 | 3561 | 4277 | + | 716 | 2 | ATT/TAA |  |
| tRNA-Arg | 4279 | 4338 | + | 59 | -2 |  | TCG |
| tRNA-His | 4336 | 4399 | + | 63 | -40 |  | GTG |
| nad5 | 4359 | 6089 | + | 1730 | 0 | ATT/TAA |  |
| tRNA-Phe | 6089 | 6149 | + | 60 | 0 |  | GAA |
| tRNA-Glu | 6149 | 6211 | + | 62 | -16 |  | TTC |
| tRNA-Pro | 6211 | 6273 | + | 62 | 3 |  | TGG |
| tRNA-Thr | 6276 | 6336 | + | 60 | -104 |  | TGT |
| nad4l | 6232 | 6612 | + | 380 | -27 | ATA/TAG |  |
| nad4 | 6585 | 7919 | + | 1334 | 6 | ATC/TAA |  |
| tRNA-Ser2 | 7925 | 7983 | + | 58 | 0 |  | TGA |
| tRNA-Ala | 7983 | 8042 | + | 59 | -1 |  | TGC |
| tRNA-Met | 8041 | 8104 | + | 63 | -2 |  | CAT |
| rrnS | 8102 | 8843 | + | 741 | -2 |  |  |
| tRNA-Gly | 8841 | 8901 | + | 60 | 5 |  | TCC |
| rrnL | 8906 | 10044 | + | 1138 | -24 |  |  |
| tRNA-Tyr | 10020 | 10081 | + | 61 | 0 |  | GTA |
| tRNA-Leu1 | 10081 | 10141 | + | 60 | 1 |  | TAG |
| tRNA-Leu2 | 10142 | 10200 | + | 58 | -48 |  | TAA |
| nad1 | 10152 | 11114 | + | 962 | 12 | ATA/TAA |  |
| tRNA-Lys | 11126 | 11187 | + | 61 | 0 |  | TTT |
| tRNA-Ile | 11187 | 11249 | + | 62 | -20 |  | GAT |
| nad3 | 11229 | 11597 | + | 368 | -1 | ATG/TAA |  |
| tRNA-Ser | 11596 | 11652 | + | 56 | -80 |  | TCT |
| nad2 | 11572 | 12606 | + | 1034 | 9 | ATG/TAA |  |
| cox1 | 12615 | 14183 | + | 1568 | 2 | ATG/TAG |  |
| tRNA-Cys | 14185 | 14244 | + | 59 | 3 |  | GCA |
| tRNA-Asn | 14247 | 14307 | + | 60 | 1 |  | GTT |
| Non coding region | 14308 | 14428 | + | 120 |  |  |  |

ovl= overlapping region, nc= non coding region
