# Supplementary figures and images for: Extensive gene rearrangements in the mitogenomes of congeneric annelid species and insights on the evolutionary history of the genus Ophryotrocha
Source: BMC Genomics. 2020 Nov 23;21:815. doi: 10.1186/s12864-020-07176-8 (PMC7682095; doi:10.1186/s12864-020-07176-8)

**Additional file 8.** tRNA structures in *O. adherens*.


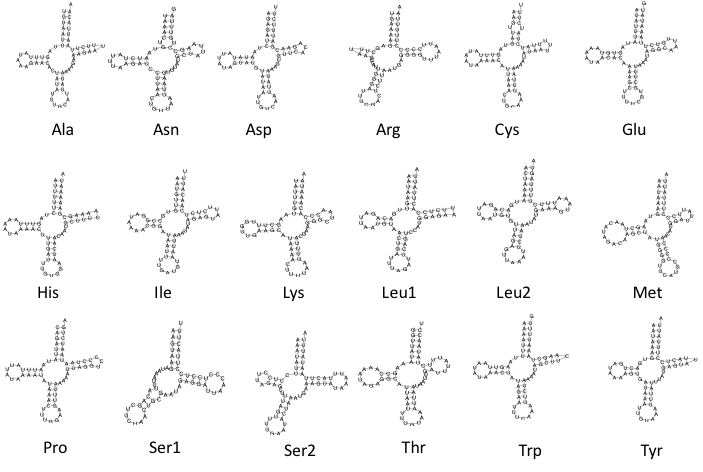

Supplement: Supplementary file 8 — Additional file 8. tRNA structures in O. adherens. [file 12864_2020_7176_MOESM8_ESM.docx]

**Additional file 9.** tRNA structures in *O. diadema*.


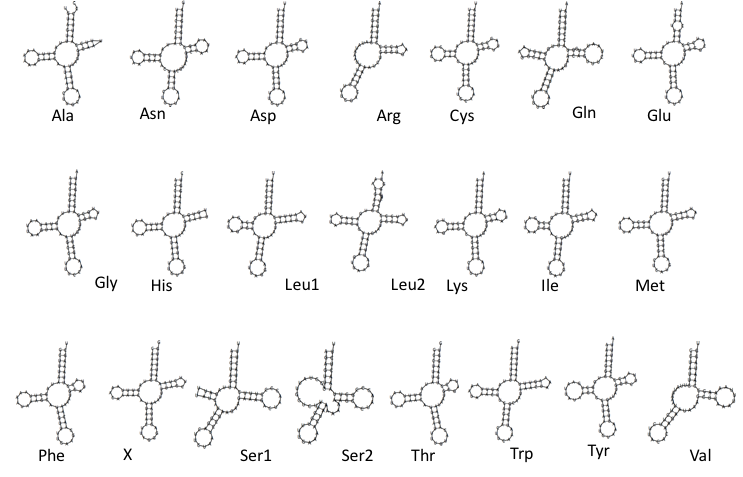

Supplement: Supplementary file 9 — Additional file 9. tRNA structures in O. diadema. [file 12864_2020_7176_MOESM9_ESM.docx]

**Additional file 10.** tRNA structures in *O. japonica*.


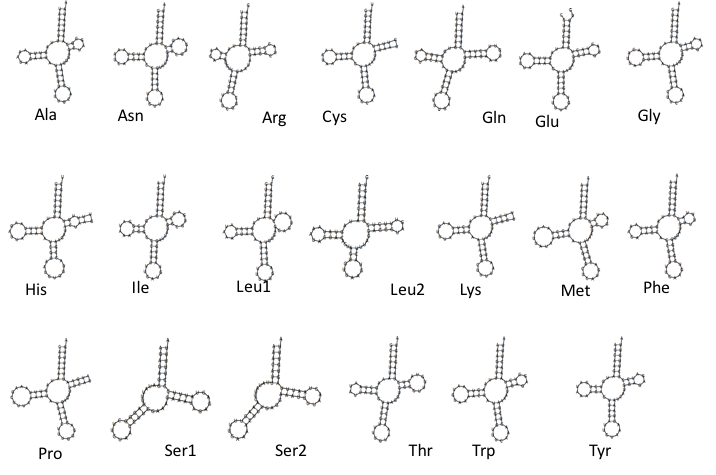

Supplement: Supplementary file 10 — Additional file 10. tRNA structures in O. japonica. [file 12864_2020_7176_MOESM10_ESM.docx]

**Additional file 11.** tRNA structures in *O. labronica*.


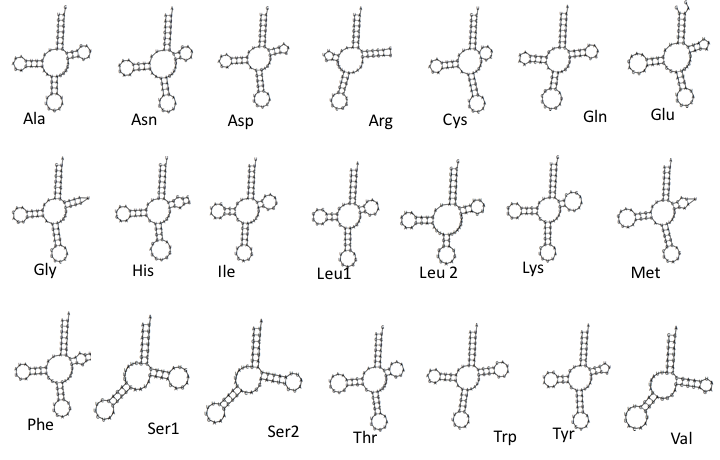

Supplement: Supplementary file 11 — Additional file 11. tRNA structures in O. labronica. [file 12864_2020_7176_MOESM11_ESM.docx]

**Additional file 12.** tRNA structures in *O. puerilis*.


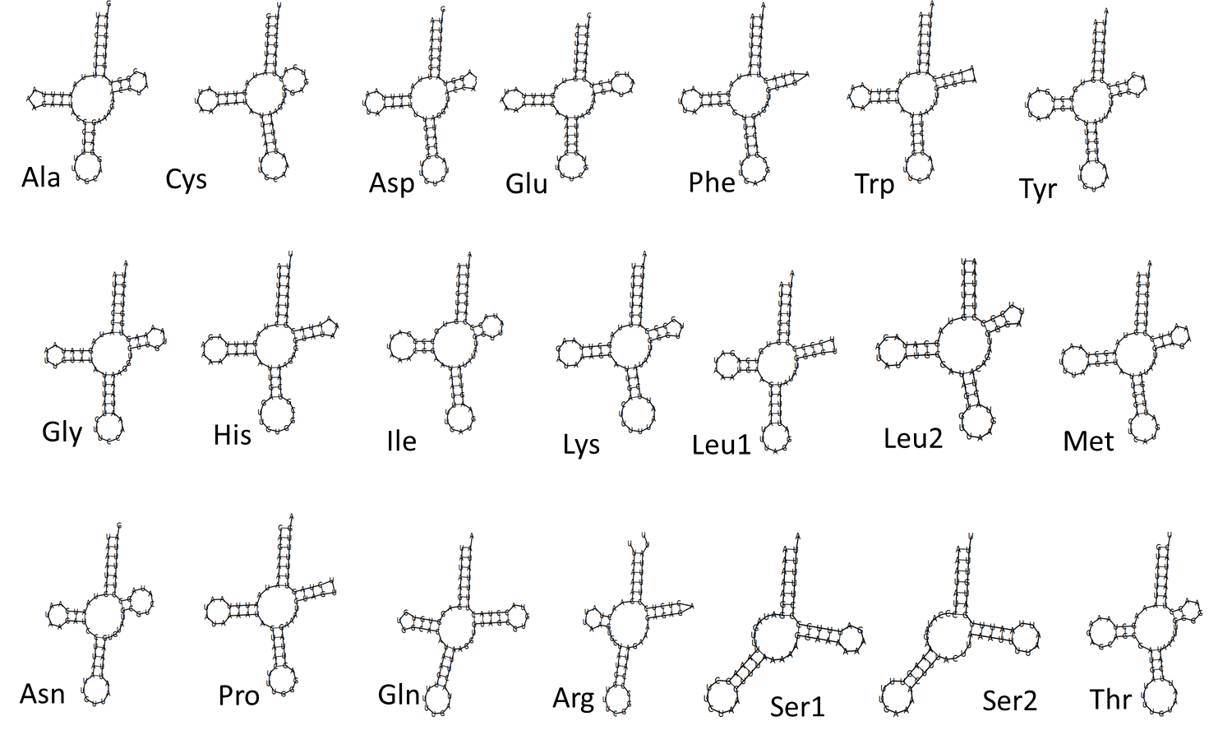

Supplement: Supplementary file 12 — Additional file 12. tRNA structures in O. puerilis. [file 12864_2020_7176_MOESM12_ESM.docx]

**Additional file 13.** tRNA structures in *O. robusta*.


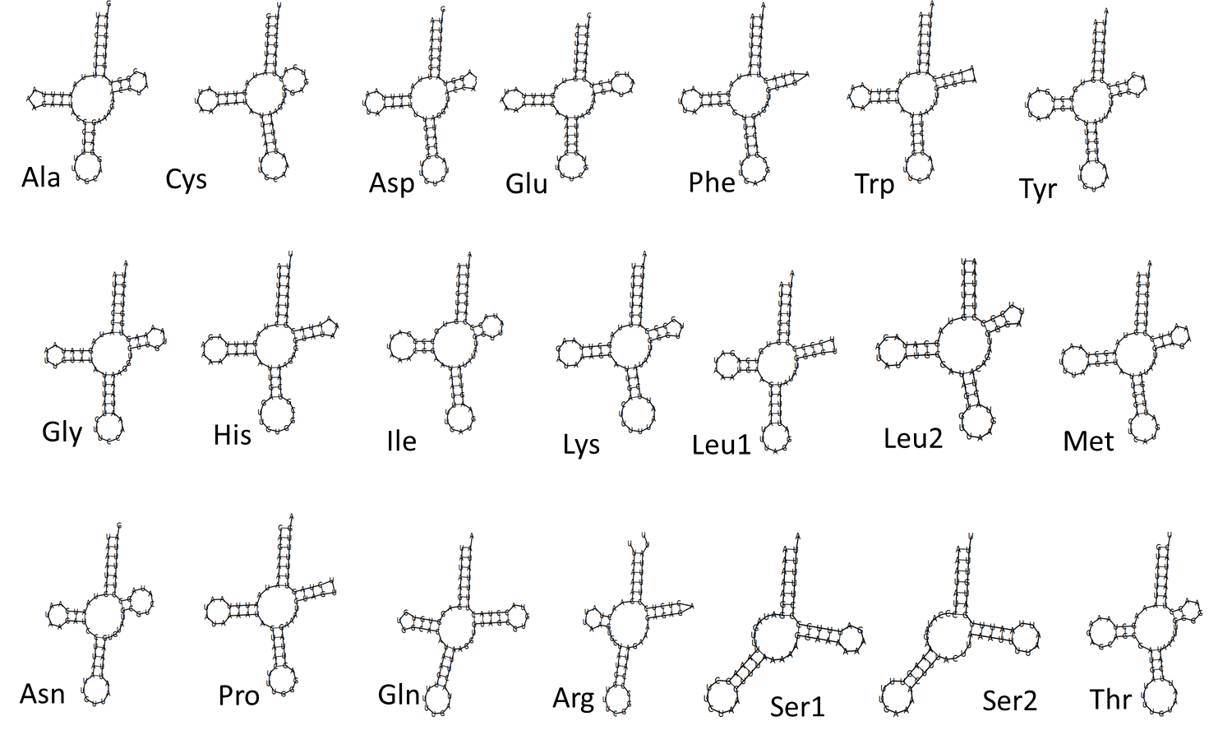

Supplement: Supplementary file 13 — Additional file 13. tRNA structures in O. robusta. [file 12864_2020_7176_MOESM13_ESM.docx]
