# Supplementary material for: Extensive gene rearrangements in the mitogenomes of congeneric annelid species and insights on the evolutionary history of the genus Ophryotrocha
Source: BMC Genomics. 2020 Nov 23;21:815. doi: 10.1186/s12864-020-07176-8 (PMC7682095; doi:10.1186/s12864-020-07176-8)
Supplement: Supplementary file 14 — Additional file 14. Matrix of the comparison between gene order of the six Ophryotrocha species investigated. [file 12864_2020_7176_MOESM14_ESM.docx]

**Additional file 14.** Matrix of the comparison between gene order of the six *Ophryotrocha* species investigated.

| Name | ***O. robusta*** | ***O. japonica*** | ***O.adherens*** | ***O. diadema*** | ***O. puerilis*** |
| --- | --- | --- | --- | --- | --- |
| ***O. robusta*** | 104 | 39 | 49 | 42 | 79 |
| ***O. japonica*** | 39 | 104 | 19 | 19 | 29 |
| ***O. adherens*** | 49 | 19 | 104 | 25 | 40 |
| ***O. diadema*** | 42 | 19 | 25 | 104 | 42 |
| ***O. puerilis*** | 79 | 29 | 40 | 42 | 104 |

The gene order of *O. labronica* was equal to *O. japonica*. Values close to 100 are mostly similar and value close to 0 are mostly dissimilar.
