# Supplementary material for: Extensive gene rearrangements in the mitogenomes of congeneric annelid species and insights on the evolutionary history of the genus Ophryotrocha
Source: BMC Genomics. 2020 Nov 23;21:815. doi: 10.1186/s12864-020-07176-8 (PMC7682095; doi:10.1186/s12864-020-07176-8)
Supplement: Supplementary file 15 — Additional file 15. Matrix of the comparison between gene order of Ophryotrocha and other annelids. [file 12864_2020_7176_MOESM15_ESM.docx]

**Additional file 15.** Matrix of the comparison between gene order of *Ophryotrocha* and other annelids.

|  | 1 | 2 | 3 | 4 | 5 | 6 | 7 | 8 | 9 | 10 | 11 | 12 | 13 | 14 | 15 | 16 | 17 | 18 | 19 |
| --- | --- | --- | --- | --- | --- | --- | --- | --- | --- | --- | --- | --- | --- | --- | --- | --- | --- | --- | --- |
| 1.Bilateral | 104 | 36 | 13 | 11 | 19 | 10 | 8 | 5 | 10 | 7 | 7 | 11 | 9 | 17 | 15 | 13 | 16 | 28 | 10 |
| 2. Magelonidae | 36 | 104 | 15 | 16 | 22 | 14 | 9 | 10 | 17 | 8 | 13 | 8 | 15 | 31 | 26 | 17 | 31 | 32 | 13 |
| 3. Oweniidae | 13 | 15 | 104 | 13 | 11 | 10 | 5 | 5 | 8 | 10 | 5 | 9 | 11 | 10 | 17 | 17 | 14 | 20 | 10 |
| 4. Cirriformidae | 11 | 16 | 13 | 104 | 18 | 17 | 15 | 13 | 24 | 9 | 19 | 9 | 22 | 40 | 47 | 40 | 55 | 12 | 14 |
| 5. Sipuncula | 19 | 22 | 11 | 18 | 104 | 15 | 13 | 9 | 11 | 9 | 13 | 10 | 23 | 30 | 24 | 24 | 32 | 13 | 17 |
| 6. Echiurida | 10 | 14 | 10 | 17 | 15 | 104 | 8 | 6 | 17 | 6 | 12 | 6 | 16 | 18 | 23 | 24 | 24 | 9 | 16 |
| 7. Trypanosyllis | 8 | 9 | 5 | 15 | 13 | 8 | 104 | 79 | 8 | 5 | 10 | 4 | 7 | 15 | 7 | 7 | 10 | 9 | 6 |
| 8. Ramisyllis | 5 | 10 | 5 | 13 | 9 | 6 | 79 | 104 | 9 | 5 | 11 | 4 | 5 | 16 | 14 | 10 | 11 | 10 | 6 |
| **9. *O. adherens*** | 10 | 17 | 8 | 24 | 11 | 17 | 8 | 9 | 104 | 5 | 25 | 7 | 19 | 38 | 44 | 40 | 49 | 15 | 15 |
| 10. Chaeptoteridae | 7 | 8 | 10 | 9 | 9 | 6 | 5 | 5 | 5 | 104 | 10 | 36 | 5 | 10 | 9 | 8 | 9 | 20 | 11 |
| **11. *O. diadema*** | 7 | 13 | 5 | 19 | 13 | 12 | 10 | 11 | 25 | 10 | 104 | 15 | 19 | 59 | 41 | 42 | 42 | 8 | 10 |
| 12. Phyllochaetopterus | 11 | 8 | 9 | 9 | 10 | 6 | 4 | 4 | 7 | 36 | 15 | 104 | 5 | 15 | 13 | 11 | 14 | 17 | 18 |
| **13. *O. japonica*** | 9 | 15 | 11 | 22 | 23 | 16 | 7 | 5 | 19 | 5 | 19 | 5 | 104 | 25 | 32 | 29 | 39 | 9 | 9 |
| 14. Ampharetidae | 17 | 31 | 10 | 40 | 30 | 18 | 15 | 16 | 38 | 10 | 59 | 15 | 25 | 104 | 68 | 58 | 79 | 23 | 14 |
| 15. Eunicidae | 15 | 26 | 17 | 47 | 24 | 23 | 7 | 14 | 44 | 9 | 41 | 13 | 32 | 68 | 104 | 91 | 91 | 18 | 17 |
| **16. *O. puerilis*** | 13 | 17 | 17 | 40 | 24 | 24 | 7 | 10 | 40 | 8 | 42 | 11 | 29 | 58 | 91 | 104 | 79 | 10 | 16 |
| 17. Pleistoannelida | 16 | 31 | 14 | 55 | 32 | 24 | 10 | 11 | 49 | 9 | 42 | 14 | 39 | 79 | 91 | 79 | 104 | 22 | 17 |
| 18. Lophotochozoa | 28 | 32 | 20 | 12 | 13 | 9 | 9 | 10 | 15 | 20 | 8 | 17 | 9 | 23 | 18 | 10 | 22 | 104 | 13 |
| 19. Eurythoe | 10 | 13 | 10 | 14 | 17 | 16 | 6 | 6 | 15 | 11 | 10 | 18 | 9 | 14 | 17 | 16 | 17 | 13 | 104 |

The gene order of *O. robusta* was identical to the gene order of Pleistoannelida. Values close to 100 are mostly similar and value close to 0 are mostly dissimilar.
