# Supplementary material for: Extensive gene rearrangements in the mitogenomes of congeneric annelid species and insights on the evolutionary history of the genus Ophryotrocha
Source: BMC Genomics. 2020 Nov 23;21:815. doi: 10.1186/s12864-020-07176-8 (PMC7682095; doi:10.1186/s12864-020-07176-8)
Supplement: Supplementary file 16 — Additional file 16. List of Ophryotrocha species used for the phylogeny. [file 12864_2020_7176_MOESM16_ESM.docx]

**Additional file 16.** List of *Ophryotrocha* species used for the phylogeny.

| **Name** | **COXI** | **16S** | **H3** | **Reproductive mode** | **Reproductive mode reference** |
| --- | --- | --- | --- | --- | --- |
| Ophryotrocha labronica #2 | MG251649 | - | - | gonochoric | 1) Paxton H. & Åkesson B. (2007). Redescription of Ophryotrocha puerilis and *O. labronica* (Annelida, Dorvilleidae). Marine Biology Research, 3: 3-19. 2) Paxton H. & Åkesson B. (2010). The *Ophryotrocha labronica* group (Annelida: Dorvilleidae) — with the description of seven new species. Zootaxa 2713: 1–24 |
| Ophryotrocha jiaolongi | KY906965 | MF398967 | MF398972 | unknown | Zhang, D. S., Zhou, Y. D., Wang, C. S., & Rouse, G. W. (2017). A new species of *Ophryotrocha* (Annelida, Eunicida, Dorvilleidae) from hydrothermal vents on the Southwest Indian Ridge. ZooKeys, (687), 1. |
| Ophryotrocha puerilis 'Shallow' | KY378520 | KY378422 | KY378638 | protandrous hermaphrodite | Paxton H. & Åkesson B. (2007). Redescription of *Ophryotrocha puerilis* and *O. labronica* (Annelida, Dorvilleidae). Marine Biology Research, 3: 3-19. |
| Ophryotrocha puerilis #2 | KY378511 | KY378420 | KY378636 | protandrous hermaphrodite |  |
| Ophryotrocha puerilis 'Deep' | KY378482 | KY378414 | KY378630 | protandrous hermaphrodite |  |
| Ophryotrocha alborana | KY378460 | KY378407 | KY378622 | simultaneous hermaphrodite | Paxton, Hannelore.; Åkesson, Bertil (2011). The *Ophryotrocha diadema* group (Annelida: Dorvilleidae), with the description of two new species. Zootaxa (3092): 43-59. |
| Ophryotrocha sp. HW | KY211995 | - | - | unknown | Taboada, S.; Wiklund, H.; Glover, A. G.; Dahlgren, T. G.; Cristobo, J.; Avila, C. (2013). Two new Antarctic Ophryotrocha (Annelida: Dorvilleidae) described from shallow-water whale bones. Polar Biology. 36(7): 1031-1045., available online at https://doi.org/10.1007/s00300-013-1326-4 |
| Ophryotrocha orensanzi | KC123178 | KC123176 | KC123180 | unknown |  |
| Ophryotocha mediterranea | KR004719 | - | - | gonochoric | Martin, Daniel, Abelló, P., and Cartes, J. (1991). A new species of Ophryotrocha (Polychaeta: Dorvilleidae) commensal in Geryon longipes (Crustacea: Brachyura) from the western Mediterranean Sea. Journal of Natural History, 25(2): 279-292. |
| **Name** | **COXI** | **16S** | **H3** | **Reproductive mode** | **Reproductive mode reference** |
| Iphitime cuenoti | KR004718 | - | - | gonochoric | Abelló, R. Sardá and D. Masa Infestation of some Mediterranean Brachyuran crabs by the Polychaete Iphitime cuenoti. Cah. Biol. Mar. (1988), 29: 149-162 |
| Ophryotrocha cyclops | KM979530 | KM979517 | KM979518 | unknown |  |
| Ophryotrocha labronica #3 | KF305814 | - | - | gonochoric | 1) Paxton H. & Åkesson B. (2007). Redescription of Ophryotrocha puerilis and *O. labronica* (Annelida, Dorvilleidae). Marine Biology Research, 3: 3-19. 2) Paxton H. & Åkesson B. (2010). The *Ophryotrocha labronica* group (Annelida: Dorvilleidae) — with the description of seven new species. Zootaxa 2713: 1–24 |
| Ophryotrocha labronica #6 | KF305781 | - | - | gonochoric |  |
| Ophryotrocha clava | KC123177 | KC123175 | KC123179 | unknown |  |
| Ophryotrocha vivipara | JQ310766 | JQ310752 | - | gonochoric | Banse, K. 1963. Polychaetous annelids from Puget Sound and the San Juan Archipelago, Washington. Proceedings of the Biological Society of Washington, 76: 197-208 2) Åkesson, B. (1994). Evolution of viviparity in the genus Ophrytrocha (Polychaeta, Dorvilleidae), in: Dauvin, J.-C. et al. (Ed.) Actes de la 4ème Conférence internationale des Polychètes, Angers, France. Mémoires du Muséum national d'Histoire naturelle. Série A, Zoologie, 162: pp. 29-35 |
| Ophryotrocha socialis | JQ310765 | AF321420 | JQ310783 | simultaneous hermaphrodite | Ockelmann, K. W.; Åkesson, B. (1990). Ophryotrocha socialis n. sp., a link between two groups of simultaneous hermaphrodites within the genus (Polychaeta, dorvilleidae). Ophelia. 31(3): 145-162., available online at https://doi.org/10.1080/00785326.1990.10430858 |
| Ophryotrocha nauarchus | JQ310764 | JQ310751 | JQ310779 | unknown |  |
| Ophryotrocha magnadentata | JQ310763 | JQ310750 | JQ310778 | unknown |  |
| Ophryotrocha macrovifera | JQ310762 | AF321430 | JQ310776 | gonochoric | Paxton, Hannelore ; Åkesson, Bertil. (2010). The Ophryotrocha labronica group (Annelida: Dorvilleidae) - with the description of seven new species. Zootaxa 2713: 1–24. , available online at http://www.mapress.com/zootaxa/2010/f/zt02713p024.pdf |
| **Name** | **COXI** | **16S** | **H3** | **Reproductive mode** | **Reproductive mode reference** |
| Ophryotrocha longicollaris | JQ310761 | JQ310749 | JQ310775 | unknown |  |
| Ophryotrocha langstrumpae | JQ310760 | JQ310748 | JQ310774 | unknown |  |
| Ophryotrocha flabella | JQ310759 | JQ310747 | JQ310772 | unknown |  |
| Ophryotrocha diadema #2 | JQ310758 | AF321425 | JQ310771 | simultaneous hermaphrodite | Paxton, Hannelore.; Åkesson, Bertil 2011. The Ophryotrocha diadema group (Annelida: Dorvilleidae), with the description of two new species. Zootaxa (3092): 43-59., available online at http://www.mapress.com/zootaxa/list/2011/3092.html |
| Ophryotrocha costlowi | JQ310757 | JQ310746 | JQ310770 | simultaneous hermaphrodite | Åkesson, B. (1976). Morphology and life cycle of Ophryotrocha diadema, a new polychaete species from California. Ophelia. 15(1): 23-35. Paxton, Hannelore.; Åkesson, Bertil 2011. The Ophryotrocha diadema group (Annelida: Dorvilleidae), with the description of two new species. Zootaxa (3092): 43-59., available online at http://www.mapress.com/zootaxa/list/2011/3092.html |
| Ophryotrocha adherens #2 | JQ310756 | AF321421 | JQ310768 | simultaneous hermaphrodite | Paavo B, Bailey-Brock JH, Åkesson B. 2000. Morphology and life history of Ophryotrocha adherens sp. nov. (Polychaeta, Dorvilleidae). Sarsia 85:251-264. |
| Protodorvillea gracilis | HM473653 | - | - | unknown |  |
| Ophryotrocha shieldsi | HM181931 | HM181932 | JQ310782 | unknown |  |
| Eunice norvegica | GQ497541 | GQ478147 | - | unknown |  |
| Ophryotrocha scutellus | GQ415488 | GQ415469 | GQ415506 | unknown |  |
| Ophryotrocha rubra #1 | GQ415487 | GQ415468 | GQ415505 | gonochoric | Paxton, Hannelore ; Åkesson, Bertil. 2010. The Ophryotrocha labronica group (Annelida: Dorvilleidae) - with the description of seven new species. Zootaxa 2713: 1–24. , available online at http://www.mapress.com/zootaxa/2010/f/zt02713p024.pdf |
| **Name** | **COXI** | **16S** | **H3** | **Reproductive mode** | **Reproductive mode reference** |
| Ophryotrocha puerilis siberti | GQ415486 | GQ415467 | GQ415504 | protandrous hermaphrodite | 1) Paxton H. & Åkesson B. (2007). Redescription of Ophryotrocha puerilis and *O. labronica* (Annelida, Dorvilleidae). Marine Biology Research, 3: 3-19. 2) Paxton H. & Åkesson B. (2010). The *Ophryotrocha labronica* group (Annelida: Dorvilleidae) — with the description of seven new species. Zootaxa 2713: 1–24 |
| Ophryotrocha puerilis puerilis | GQ415485 | GQ415466 | GQ415503 | protandrous hermaphrodite |  |
| Ophryotrocha permanni #1 | GQ415484 | AF321432 | GQ415502 | gonochoric | Heggoy et al., 2007 |
| Ophryotrocha maculata | GQ415483 | GQ415465 | JQ310777 | simultaneous hermaphrodite | Åkesson, B. 1973. Morphology and life history of Ophryotrocha maculata sp.n. (Polychaeta, Dorvilleidae). Zoologica Scripta, 2: 141-144 |
| Ophryotrocha longidentata | GQ415482 | GQ415471 | GQ415501 | unknown |  |
| Ophryotrocha labronica #7 | GQ415479 | GQ415463 | GQ415499 | gonochoric | 1) Paxton H. & Åkesson B. (2007). Redescription of Ophryotrocha puerilis and *O. labronica* (Annelida, Dorvilleidae). Marine Biology Research, 3: 3-19. 2) Paxton H. & Åkesson B. (2010). The *Ophryotrocha labronica* group (Annelida: Dorvilleidae) — with the description of seven new species. Zootaxa 2713: 1–24 |
| Ophryotrocha japonica #2 | GQ415478 | GQ415462 | GQ415496 | gonochoric | Paxton, Hannelore ; Åkesson, Bertil. 2010. The Ophryotrocha labronica group (Annelida: Dorvilleidae) - with the description of seven new species. Zootaxa 2713: 1–24. , page(s): 7 ; Prevedelli, D.; Massamba N'Siala, G.; Simonini, R. (2005). The seasonal dynamics of six species of Dorvilleidae (Polychaeta) in the harbour of La Spezia (Italy). Marine Ecology. 26(3-4): 286-293; Simonini, R. (2002). Distribution and ecology of the genus Ophryotrocha (Polychaeta: Dorvilleidae) in Italian harbours and lagoons. Vie Milieu. 52: 59-65. available online at http://www.mapress.com/zootaxa/2010/f/zt02713p024.pdf |
| Ophryotrocha globopalpata | GQ415477 | GQ415461 | GQ415495 | unknown |  |
| **Name** | **COXI** | **16S** | **H3** | **Reproductive mode** | **Reproductive mode reference** |
| Ophryotrocha geryonicola | GQ415476 | GQ415460 | GQ415494 | gonochoric | Pfannenstiel, H.D., Grothe, C. & Kegel, B. Helgolander Meeresunters (1982) 35: 119. https://doi.org/10.1007/BF02289838. Studies on Ophryotrocha geryonicola (Polychaeta: Dorvilleidae) |
| Ophryotrocha eutrophila | GQ415475 | GQ415459 | GQ415493 | gonochoric | Wiklund, H., Glover, A. G., & Dahlgren, T. G. (2009). Three new species of Ophryotrocha (Annelida: Dorvilleidae) from a whale-fall in the North-East Atlantic. Zootaxa, 2228(1), 43-56. |
| Ophryotrocha craigsmithi | GQ415474 | - | GQ415491 | unknown |  |
| Iphitime hartmanae | GQ415472 | GQ415458 | - | unknown |  |
| Iphitime paguri | EF464549 | - | - | gonochoric | TORE HØISÆTER & TOR J. SAMUELSEN Taxonomic and biological notes on a species of Iphitime (Polychaeta, Eunicida) associated with Pagurus prideaux from western Norway. Marine Biology Research, 2006; 2: 333 354 |
| Ophryotrocha sp. Benidorm | EF464548 | - | - | unknown |  |
| Ophryotrocha robusta #2 | EF464547 | AF321433 | JQ310781 | gonochoric | Paxton H. & Åkesson B. (2010). The Ophryotrocha labronica group (Annelida: Dorvilleidae) with the description of seven new species. Zootaxa 2713: 1–24 |
| Ophryotrocha hartmanni | EF464546 | AF321419 | JQ310773 | simultaneous hermaphrodite | Prevedelli, D.; Massamba N'Siala, G.; Simonini, R. (2005). The seasonal dynamics of six species of Dorvilleidae (Polychaeta) in the harbour of La Spezia (Italy). Marine Ecology. 26(3-4): 286-293. |
| Ophryotrocha gracilis | EF464545 | AF321424 | GQ415497 | simultaneous hermaphrodite | Parenti, U. 1964. Inversione sessuale limitata a due metameri in Ophryotrocha gracilis. Bollettino di Zoologia 31: 25-31 |
| Ophryotrocha puerilis | EF464544 | - | - | protandrous hermaphrodite | 1) Paxton H. & Åkesson B. (2007). Redescription of Ophryotrocha puerilis and *O. labronica* (Annelida, Dorvilleidae). Marine Biology Research, 3: 3-19. 2) Paxton H. & Åkesson B. (2010). The *Ophryotrocha labronica* group (Annelida: Dorvilleidae) — with the description of seven new species. Zootaxa 2713: 1–24 |
| **Name** | **COXI** | **16S** | **H3** | **Reproductive mode** | **Reproductive mode reference** |
| Ophryotrocha rubra #2 | EF464543 | - | - | gonochoric | Paxton H. & Åkesson B. (2010). The Ophryotrocha labronica group (Annelida: Dorvilleidae) with the description of seven new species. Zootaxa 2713: 1–24 |
| Ophryotrocha notoglandulata | EF464542 | - | JQ310780 | gonochoric |  |
| Ophryotrocha japonica #3 | EF464541 | - | GQ415498 | gonochoric | Paxton, Hannelore ; Åkesson, Bertil. 2010. The Ophryotrocha labronica group (Annelida: Dorvilleidae) - with the description of seven new species. Zootaxa 2713: 1–24. , available online at http://www.mapress.com/zootaxa/2010/f/zt02713p024.pdf |
| Ophryotrocha sp. Qingdao | EF464539 | - | AF321426 | simultaneous hermaphrodite | Dahlgren et al., 2001; Heggøy et al. 2007 |
| Ophryotrocha sp. Eilat-Hurgada | EF464538 | AF321431 | AF321436 | gonochoric |  |
| Ophryotrocha sp. Sanya | EF464537 | GQ415463 | AF321434 | gonochoric |  |
| Ophryotrocha labronica #5 | EF464536 | AF321426 | - | gonochoric | 1) Paxton H. & Åkesson B. (2007). Redescription of Ophryotrocha puerilis and *O. labronica* (Annelida, Dorvilleidae). Marine Biology Research, 3: 3-19. 2) Paxton H. & Åkesson B. (2010). The *Ophryotrocha labronica* group (Annelida: Dorvilleidae) — with the description of seven new species. Zootaxa 2713: 1–24 |
| Ophryotrocha permanni #2 | EF464535 | AF321436 | - |  | Heggoy et al., 2007 |
| Ophryotrocha diadema #3 | EF464534 | AF321434 | - | simultaneous hermaphrodite | original description Åkesson, B. (1976). Morphology and life cycle of Ophryotrocha diadema, a new polychaete species from California. Ophelia. 15(1): 23-35. Paxton, Hannelore.; Åkesson, Bertil 2011. The Ophryotrocha diadema group (Annelida: Dorvilleidae), with the description of two new species. Zootaxa (3092): 43-59., available online at http://www.mapress.com/zootaxa/list/2011/3092.html |
| Ophryotrocha robusta | MT737361 | MT737361 | MT733538 | gonochoric | Paxton H. & Åkesson B. (2010). The Ophryotrocha labronica group (Annelida: Dorvilleidae) with the description of seven new species. Zootaxa 2713: 1–24 |
| **Name** | **COXI** | **16S** | **H3** | **Reproductive mode** | **Reproductive mode reference** |
| Ophryotrocha puerilis | MT737365 | MT737365 | MT733539 | protandrous hermaphrodite | Paxton H. & Åkesson B. (2007). Redescription of *Ophryotrocha puerilis* and *O. labronica* (Annelida, Dorvilleidae). Marine Biology Research, 3: 3-19. |
| Ophryotrocha labronica | MT737362 | MT737362 | MT733540 | gonochoric | 1) Paxton H. & Åkesson B. (2007). Redescription of Ophryotrocha puerilis and *O. labronica* (Annelida, Dorvilleidae). Marine Biology Research, 3: 3-19. 2) Paxton H. & Åkesson B. (2010). The *Ophryotrocha labronica* group (Annelida: Dorvilleidae) — with the description of seven new species. Zootaxa 2713: 1–24 |
| Ophryotrocha japonica | MT7373633 | MT737363 | MT733541 | gonochoric | Paxton, Hannelore ; Åkesson, Bertil. 2010. The Ophryotrocha labronica group (Annelida: Dorvilleidae) - with the description of seven new species. Zootaxa 2713: 1–24. , available online at http://www.mapress.com/zootaxa/2010/f/zt02713p024.pdf |
| Ophryotrocha diadema | MT737364 | MT737364 | MT733542 | simultaneous hermaphrodite | original description Åkesson, B. (1976). Morphology and life cycle of Ophryotrocha diadema, a new polychaete species from California. Ophelia. 15(1): 23-35. Paxton, Hannelore.; Åkesson, Bertil 2011. The Ophryotrocha diadema group (Annelida: Dorvilleidae), with the description of two new species. Zootaxa (3092): 43-59., available online at http://www.mapress.com/zootaxa/list/2011/3092.html |
| Ophryotrocha adherens | MT737360 | MT737360 | MT733543 | simultaneous hermaphrodite | Paavo B, Bailey-Brock JH, Åkesson B. 2000. Morphology and life history of Ophryotrocha adherens sp. nov. (Polychaeta, Dorvilleidae). Sarsia 85:251-264. |
| Ophryotrocha labronica #4 | AY838874 | AF321429 | - | gonochoric | 1) Paxton H. & Åkesson B. (2007). Redescription of Ophryotrocha puerilis and *O. labronica* (Annelida, Dorvilleidae). Marine Biology Research, 3: 3-19. 2) Paxton H. & Åkesson B. (2010). The *Ophryotrocha labronica* group (Annelida: Dorvilleidae) — with the description of seven new species. Zootaxa 2713: 1–24 |
| **Name** | **COXI** | **16S** | **H3** | **Reproductive mode** | **Reproductive mode reference** |
| Eunice pennata | AY838870 | AF321418 | DQ779731 | unknown |  |
| Ophryotrocha obscura | - | AF321435 | - | unknown |  |
| Ophryotrocha sadina | - | - | KP731533 | unknown |  |
| Ophryotrocha mammillata | - | - | KP731524 | unknown |  |
| Ophryotrocha lusa | - | - | KP731500 | unknown |  |
| Ophryotrocha batillus | - | JQ310745 | JQ310769 | unknown |  |
| Dorvillea erucaeformis | AY838868 | AY838827 | - | unknown |  |
