# Supplementary material for: Extensive gene rearrangements in the mitogenomes of congeneric annelid species and insights on the evolutionary history of the genus Ophryotrocha
Source: BMC Genomics. 2020 Nov 23;21:815. doi: 10.1186/s12864-020-07176-8 (PMC7682095; doi:10.1186/s12864-020-07176-8)
Supplement: Supplementary file 17 — Additional file 17. Ancestral reproductive mode reconstruction based on Bayesian posterior probabilities. [file 12864_2020_7176_MOESM17_ESM.docx]

**Additional file 17.** List of the annelid species used in the study.

| **Name** | **Class** | **Status** | **Length** | **Accession Number** |
| --- | --- | --- | --- | --- |
| 1. *Cheilonereis cyclurus* | Errantia | complete genome | 14,971 bp | MF538532.1 |
| 2. *Nereis sp.* | Errantia | complete genome | 15,667 bp | MF960765.1 |
| 3. *Cirriformia cf. tentaculata* HK-2018 | Sedentaria | complete genome | 15,495 bp | NC_037390.1 |
| 4. *Cryptonome barbada* | Errantia | complete genome | 15,013 bp | NC_037947.1 |
| 5. *Spirobranchus giganteus* | Sedentaria | complete genome | 22,058 bp | NC_032055.1 |
| 6. *Sipunculus nudus* | Sipuncula | complete genome | 15,375 bp | MG873457.1 |
| 7. *Pholoe pallida* | Errantia | partial genome | 15,473 bp | KY753838.1 |
| 8. *Euthalenessa festiva* | Errantia | partial genome | 15,023 bp | KY753837.1 |
| 9. *Pisione sp*. YZ-2018 | Errantia | partial genome | 15,394 bp | KY753836.1 |
| 10. *Iphione sp.* YZ-2018 | Errantia | partial genome | 15,385 bp | KY753835.1 |
| 11. *Eulepethus nanhaiensis* | Errantia | partial genome | 15,351 bp | KY753834.1 |
| 12. *Laetmonice producta* | Errantia | partial genome | 15,677 bp | KY753833.1 |
| 13. *Panthalis oerstedi* | Errantia | partial genome | 15,261 bp | KY753832.1 |
| 14. *Lepidonotus sp.* YZ-2018 | Errantia | partial genome | 15,134 bp | KY753831.1 |
| 15. *Halosydna sp.* YZ-2018 | Errantia | partial genome | 15,124 bp | KY753830.1 |
| 16. *Melaenis sp.* YZ-2018 | Errantia | partial genome | 15,164 bp | KY753829.1 |
| 17. *Lepidonotopodium sp.* YZ-2018 | Errantia | partial genome | 16,373 bp | KY753828.1 |
| 18. *Levensteiniella iris* | Errantia | partial genome | 16,036 bp | KY753827.1 |
| 19. *Branchipolynoe sp.* YZ-2018 | Errantia | partial genome | 15,968 bp | KY753826.1 |
| 20. *Branchipolynoe pettiboneae* | Errantia | partial genome | 15,202 bp | KY753825.1 |
| 21. *Branchinotogluma japonicus* | Errantia | partial genome | 15,513 bp | KY753824.1 |
| 22. *Marphysa tamurai* | Errantia | complete genome | 15,163 bp | NC_037236.1 |
| 23. *Paraescarpia echinospica* | Sedentaria | complete genome | 15,280 bp | NC_037085.1 |
| 24. *Dinophilus gyrociliatus* | probably Errantia | partial genome | 14,678 bp | MG428625.1 |
| 25. *Erpobdella japonica* | Clitellata | complete genome | 14,725 bp | NC_036150.1 |
| 26. *Neanthes glandicincta* | Errantia | complete genome | 16,126 bp | NC_035893.1 |
| 27. *Hediste diadroma* | Errantia | complete genome | 15,765 bp | NC_035507.1 |
| 28. *Zeylanicobdella arugamensis* | Clitellata | complete genome | 16,161 bp | NC_035308.1 |
| 29. *Decemunciger sp.* AB-2017 voucher A3372-2 | Sedentaria | complete genome | 19,003 bp | KY774371.1 |
| 30. *Decemunciger sp.* AB-2017 voucher A3372-1 | Sedentaria | complete genome | 19,096 bp | KY774370.1 |
| 31. *Decemunciger sp.* AB-2017 | Sedentaria | complete genome | 19,274 bp | KY742027.1 |
| 32. *Ozobranchus jantseanus* | Clitellata | complete genome | 14,864 bp | NC_034807.1 |
| 33. *Pontoscolex corethrurus* | Clitellata | complete genome | 14,835 bp | NC_034783.1 |
| 34. *Phascolosoma sp.* MZK-2017 | Sipuncula | partial genome | 16,571 bp | KX814447.1 |
| 35. *Paraleonnates uschakovi* | Errantia | complete genome | 15,540 bp | NC_032361.1 |
| 36. *Phascolosoma pacificum* | Sipuncula | complete genome | 16,039 bp | NC_031412.1 |
| 37. *Myrianida brachycephala* | Errantia | complete genome | 15,032 bp | NC_031403.1 |
| 38. *Eusyllis blomstrandi* | Errantia | complete genome | 14,712 bp | NC_031402.1 |
| 39. *Typosyllis antoni* | Errantia | complete genome | 16,897 bp | NC_031404.1 |
| 40. *Hirudo nipponia* | Clitellata | complete genome | 14,414 bp | NC_023776.1 |
| 41. *Endomyzostoma sp.* MZ-2009 | probably Errantia | partial genome | 13,190 bp | FJ975144.1 |
| 42. *Questa ersei* voucher | Sedentaria | partial genome | 11,909 bp | FJ612452.1 |
| 43. *Paralvinella sulfincola* | Sedentaria | partial genome | 13,640 bp | FJ976042.1 |
| 44. *Auchenoplax crinita* | Sedentaria | partial genome | 13,759 bp | FJ976041.1 |
| 45. *Pectinaria gouldii* | Sedentaria | partial genome | 13,438 bp | FJ976040.1 |
| 46. *Myzostoma seymourcollegiorum* | probably Errantia | partial genome | 11,505 bp | EF506562.1 |
| 47. *Laeonereis culveri* | Errantia | complete genome | 14,918 bp | KU992689.1 |
| 48. *Metaphire vulgaris* | Clitellata | complete genome | 15,061 bp | NC_023836.1 |
| 49. *Placobdella lamothei* strain MXTON | Clitellata | complete genome | 15,190 bp | NC_030269.1 |
| 50. *Namalycastis abiuma* | Errantia | complete genome | 15,265 bp | NC_030040.1 |
| 51. *Scoloplos cf. armiger* CB-2006 | Sedentaria | partial genome | 12,042 bp | DQ517436.1 |
| 52. *Placobdella parasitica* strain ONAP | Clitellata | partial genome | 14,909 bp | LT159850.1 |
| 53. *Haementeria officinalis* strain GTOCOR | Clitellata | partial genome | 14,849 bp | LT159848.1 |
| 54. *Hirudo verbana* | Clitellata | partial genome | 14,604 bp | KU672397.1 |
| 55. *Hirudo medicinalis* | Clitellata | partial genome | 14,729 bp | KU672396.1 |
| 56. *Amynthas jiriensis* | Clitellata | complete genome | 15,151 bp | NC_029879.1 |
| 57. *Amynthas moniliatus* | Clitellata | complete genome | 15,133 bp | NC_029872.1 |
| 58. *Amynthas robustus* | Clitellata | complete genome | 15,013 bp | NC_029871.1 |
| 59. *Amynthas pectiniferus* | Clitellata | complete genome | 15,188 bp | NC_029870.1 |
| 60. *Metaphire guillelmi* | Clitellata | complete genome | 15,174 bp | NC_029869.1 |
| 61. *Amynthas triastriatus* | Clitellata | complete genome | 15,160 bp | NC_029868.1 |
| 62. *Duplodicodrilus schmardae* | Clitellata | complete genome | 15,156 bp | NC_029867.1 |
| 63. *Amynthas cucullatus* | Clitellata | complete genome | 15,122 bp | NC_029866.1 |
| 64. *Amynthas morrisi* | Clitellata | complete genome | 15,026 bp | NC_029865.1 |
| 65. *Amynthas hupeiensis* | Clitellata | complete genome | 15,069 bp | NC_029864.1 |
| 66. *Amynthas carnosus* | Clitellata | complete genome | 15,160 bp | NC_029863.1 |
| 67. *Amynthas sp. 2* LZ-2016 | Clitellata | complete genome | 15,086 bp | KT429014.1 |
| 68. *Amynthas sp. 3* LZ-2016 | Clitellata | complete genome | 15,152 bp | KT429013.1 |
| 69. *Amynthas sp. 1* LZ-2016 | Clitellata | complete genome | 15,131 bp | KT429010.1 |
| 70. *Amynthas sp. 2* JS-2012 | Clitellata | complete genome | 15,159 bp | KT429007.1 |
| 71. *Sipunculus nudus* | Sipuncula | complete genome | 15,303 bp | KP751904.1 |
| 72. *Eurythoe complanata* | Errantia | complete genome | 15,829 bp | NC_028714.1 |
| 73. *Owenia fusiformis* | Sedentaria | complete genome | 16,204 bp | NC_028712.1 |
| 74. *Magelona mirabilis* | probably Sedentaria | complete genome | 15,239 bp | NC_028711.1 |
| 75. *Chaetopterus variopedatus* | Sedentaria | complete genome | 16,143 bp | NC_028710.1 |
| 76. *Glycera cf. oxycephala* FS21 | Errantia | partial genome | 15,852 bp | KT989329.1 |
| 77. *Glycinde armigera* isolate FS17 | Errantia | partial genome | 15,077 bp | KT989325.1 |
| 78. *Glycera fallax* isolate FS14 | Errantia | partial genome | 20,896 bp | KT989323.1 |
| 79. *Glycera americana* isolate FS12 | Errantia | partial genome | 15,571 bp | KT989321.1 |
| 80. *Glycera tridactyla* isolate Glytri | Errantia | partial genome | 15,361 bp | KT989331.1 |
| 81. *Glycera cf. tridactyla* FS20 | Errantia | complete genome | 15,510 bp | KT989328.1 |
| 82. *Glycera cf. tridactyla* FS19 | Errantia | complete genome | 15,373 bp | KT989327.1 |
| 83. *Glycera tesselata* isolate FS18 | Errantia | complete genome | 15,591 bp | KT989326.1 |
| 84. *Hemipodia simplex* isolate FS13 | Errantia | complete genome | 15,401 bp | KT989322.1 |
| 85. *Glycera capitata* isolate FS11 | Errantia | complete genome | 15,178 bp | KT989320.1 |
| 86. *Glycera capitata* isolate FS10 | Errantia | complete genome | 15,182 bp | KT989319.1 |
| 87. *Glycera dibranchiata* isolate FS05 | Errantia | complete genome | 16,085 bp | KT989318.1 |
| 88. *Whitmania laevis* | Clitellata | complete genome | 14,442 bp | KM655839.1 |
| 89. *Phyllochaetopterus sp.* AW-2015 | Sedentaria | complete genome | 16,087 bp | KT726961.1 |
| 90. *Drawida japonica* | Clitellata | complete genome | 14,648 bp | NC_028050.1 |
| 91. *Lamellibrachia satsuma* | Sedentaria | complete genome | 15,037 bp | NC_027854.1 |
| 92. *Amynthas corticis* | Clitellata | complete genome | 15,126 bp | NC_027832.1 |
| 93. *Amynthas longisiphonus* | Clitellata | complete genome | 15,176 bp | NC_027831.1 |
| 94. *Ramisyllis multicaudata* | Errantia | complete genome | 15,748 bp | NC_027699.1 |
| 95. *Trypanosyllis* (Trypanobia) *sp.* CB-2015 | Errantia | complete genome | 16,630 bp | KR534503.1 |
| 96. *Amynthas gracilis* | Clitellata | complete genome | 15,161 bp | NC_027258.1 |
| 97. *Metaphire californica* | Clitellata | complete genome | 15,147 bp | NC_027257.1 |
| 98. *Goniada japonica* | Errantia | complete genome | 15,327 bp | NC_026995.1 |
| 99. *Tevnia jerichonana* | Sedentaria | complete genome | 14,891 bp | NC_026862.1 |
| 100. *Oasisia alvinae* | Sedentaria | complete genome | 14,849 bp | NC_026859.1 |
| 101. *Escarpia spicata* | Sedentaria | complete genome | 15,445 bp | NC_026856.1 |
| 102. *Seepiophila jonesi* | Sedentaria | complete genome | 15,092 bp | NC_026861.1 |
| 103. *Riftia pachyptila* | Sedentaria | complete genome | 14,987 bp | NC_026860.1 |
| 104. *Lamellibrachia luymesi* | Sedentaria | complete genome | 14,991 bp | NC_026858.1 |
| 105. *Galathealinum brachiosum* | Sedentaria | complete genome | 14,779 bp | NC_026857.1 |
| 106. *Sclerolinum brattstromi* | Sedentaria | complete genome | 15,383 bp | NC_026855.1 |
| 107. *Siboglinum fiordicum* | Sedentaria | complete genome | 19,502 bp | NC_026833.1 |
| 108. *Spirobrachia sp.* YL-2014 | Sedentaria | complete genome | 15,581 bp | KJ789171.1 |
| 109. *Siboglinum ekmani* | Sedentaria | partial genome | 14,838 bp | KJ789169.1 |
| 110. *Ridgeia piscesae* | Sedentaria | partial genome | 14,146 bp | KJ789165.1 |
| 111. *Tylorrhynchus heterochaetus* | Errantia | complete genome | 16,106 bp | NC_025561.1 |
| 112. *Amynthas aspergillu*s isolate M01 | Clitellata | complete genome | 15,115 bp | NC_025292.1 |
| 113. *Ridgeia piscesae* | Sedentaria | complete genome | 15,002 bp | NC_024653.1 |
| 114. *Sipunculus nudus* | Sipuncula | complete genome | 15,376 bp | KJ754934.1 |
| 115. *Lumbricus terrestris* | Clitellata | complete genome | 14,998 bp | NC_001673.1 |
| 116. *Whitmania laevis* | Clitellata | complete genome | 14,433 bp | NC_023926.1 |
| 117. *Poecilobdella manillensis* | Clitellata | complete genome | 14,470 bp | NC_023925.1 |
| 118. *Perinereis aibuhitensis* | Errantia | complete genome | 15,852 bp | NC_023943.1 |
| 119. *Whitmania acranulata* | Clitellata | complete genome | 14,462 bp | NC_023928.1 |
| 120. *Erpobdella octoculata* | Clitellata | complete genome | 14,407 bp | NC_023927.1 |
| 121. *Hirudinaria manillensis* | Clitellata | complete genome | 14,470 bp | KC688268.1 |
| 122. *Marphysa sanguinea* | Errantia | complete genome | 15,159 bp | NC_023124.1 |
| 123. *Tonoscolex birmanicus* | Clitellata | complete genome | 15,170 bp | KF425518.1 |
| 124. *Perinereis nuntia* | Errantia | complete genome | 15,824 bp | NC_020609.1 |
| 125. *Diurodrilus subterraneus* | probably Errantia | partial genome | 13,025 bp | KC790350.1 |
| 126. *Whitmania pigra* | Clitellata | complete genome | 14,426 bp | NC_013569.1 |
| 127. *Urechis unicinctus* | Sedentaria | complete genome | 15,761 bp | NC_012768.1 |
| 128. *Orbinia latreillii* | Sedentaria | complete genome | 15,558 bp | NC_007933.1 |
| 129. *Urechis caupo* | Sedentaria | complete genome | 15,113 bp | NC_006379.1 |
| 130. *Clymenella torquata* | Sedentaria | complete genome | 15,538 bp | NC_006321.1 |
| 131. *Platynereis dumerilii* | Errantia | complete genome | 15,619 bp | NC_000931.1 |
| 132. *Phascolosoma esculenta* | Sipuncula | complete genome | 15,494 bp | NC_012618.1 |
| 133. *Sipunculus nudus* | Sipuncula | complete genome | 15,502 bp | NC_011826.1 |
| 134. *Pista cristata* | Sedentaria | complete genome | 15,894 bp | NC_011011.1 |
| 135. *Terebellides stroemii* | Sedentaria | complete genome | 15,755 bp | NC_011014.1 |
| 136. *Nephtys sp.* 'San Juan Island' YV-2008 | Errantia | complete genome | 17,217 bp | NC_010559.1 |
| 137. *Perionyx excavatus* | Clitellata | complete genome | 15,083 bp | NC_009631.1 |
| 138. *Eclysippe vanelli* | Sedentaria | partial genome | 13,749 bp | EU239687.1 |
